# Complete loss of miR-200 family induces EMT associated cellular senescence in gastric cancer

**DOI:** 10.1038/s41388-021-02067-y

**Published:** 2021-10-19

**Authors:** Liang Yu, Can Cao, Xu Li, Mili Zhang, Qisheng Gu, Hugh Gao, Jesse J. Balic, Duogang Xu, Lei Zhang, Le Ying, Dakang Xu, Yuqin Yang, Di Wu, Baokun He, Brendan J. Jenkins, Youdong Liu, Jikun Li

**Affiliations:** 1 Department of General Surgery, Shanghai General Hospital, Shanghai Jiao Tong University School of Medicine, Shanghai, 200080 China; 2 Institut Pasteur of Shanghai, Chinese Academy of Sciences, Shanghai, 200031 China; 3 Department of Clinical Pathology, University of Melbourne, Melbourne, Victoria, 3133 Australia; 4 Department of Upper Gastrointestinal and Hepatobiliary Surgery, Monash Health, Clayton, Victoria, 3168 Australia; 5 Centre for Innate Immunity and Infectious Diseases, Hudson Institute of Medical Research, Clayton, Victoria, 3168 Australia; 6 Department of Molecular Translational Science, Faculty of Medicine, Nursing and Health Sciences, Monash University, Clayton, Victoria, 3800 Australia; 7 Shanghai Key Laboratory of Pancreatic Disease, Shanghai General Hospital, Shanghai Jiao Tong University School of Medicine, Shanghai, 201620 China; 8 Faculty of Medical Laboratory Science, Ruijin Hospital, School of Medicine, Shanghai Jiao Tong University, Shanghai, China; 9 Department of Laboratory Animal Centre, Shanghai General Hospital, Shanghai Jiao Tong University School of Medicine, Shanghai, 201620 China; 10 Department of Biostatistics, UNC Gillings School of Global Public Health, University of North Carolina, Chapel Hill, USA

**Keywords:** Cancer microenvironment, Gastric cancer, Senescence

## Abstract

The EMT (epithelial-to-mesenchymal-transition) subtype of gastric cancer (GC) is associated with poor treatment responses and unfavorable clinical outcomes. Despite the broad physiological roles of the micro-RNA (miR)-200 family, they largely serve to maintain the overall epithelial phenotype. However, during late-stage gastric tumorigenesis, members of the miR-200 family are markedly suppressed, resulting in the transition to the mesenchymal state and the acquisition of invasive properties. As such, the miR-200 family represents a robust molecular marker of EMT, and subsequently, disease severity and prognosis. Most reports have studied the effect of single miR-200 family member knockdown. Here, we employ a multiplex CRISPR/Cas9 system to generate a complete miR-200 family knockout (FKO) to investigate their collective and summative role in regulating key cellular processes during GC pathogenesis. Genetic deletion of all miR-200s in the human GC cell lines induced potent morphological alterations, G1/S cell cycle arrest, increased senescence-associated β-galactosidase (SA-β−Gal) activity, and aberrant metabolism, collectively resembling the senescent phenotype. Coupling RNA-seq data with publicly available datasets, we revealed a clear separation of senescent and non-senescent states amongst FKO cells and control cells, respectively. Further analysis identified key senescence-associated secretory phenotype (SASP) components in FKO cells and a positive feedback loop for maintenance of the senescent state controlled by activation of TGF-β and TNF-α pathways. Finally, we showed that miR-200 FKO associated senescence in cancer epithelial cells significantly recruited stromal cells in the tumor microenvironment. Our work has identified a new role of miR-200 family members which function as an integrated unit serving to link senescence with EMT, two major conserved biological processes.

## Introduction

The prognosis of gastric cancer (GC) patients, the third leading cause of cancer-related death, is largely dictated by the stage of the disease identified at diagnosis. Despite the advances of adjunctive therapies, patients with late-stage GC that are receiving surgery, chemotherapy, and immunotherapy in the (neo)adjuvant setting still experience significant treatment resistance, relapse, and metastasis [1]. Much effort has focused on comprehensively performing genome-wide surveys to identify the underlying molecular mechanisms that drive GC aggressiveness and malignancy [2]. One of the molecular subtypes of GC which is a predictor of poor treatment responses and unfavorable survival in GC with a mesenchymal/EMT phenotype [3]. Recent studies also demonstrate that the EMT subtype is associated with an immune suppressive tumor-microenvironment, resulting in cancer cell evasion and persistence, and subsequently, poor responses to PD-1-based immunotherapies [1, 4].

The miR-200 family of microRNAs has been well identified as key regulations of specifying and maintaining the epithelial phenotype by inhibiting the expression of the transcription factors, ZEB1 and ZEB2 [5, 6]. Loss of miR-200s in various human cancers is associated with a mesenchymal/EMT cancer type and associated with poor therapeutic outcomes [7, 8]. The expression level of five miR-200 family members in GC is often highly inversely correlated with cancer progression and indeed is strongly downregulated in the late stage of disease [9]. Interestingly, all five of the miR-200 family members are found to be downregulated, despite these molecules existing across two separate genomic loci. Currently, it is unclear how to complete loss of the miR-200 family affects EMT in the context of GC.

In this study, we utilized a multiplex CRISPR/Cas9 system to generate single-cell clones with complete or partial miR-200 family knockout in the commonly used GC cell lines. Loss of all miR-200s (FKO) in GC cells induced cytoskeletal and cell junction disruptions. Importantly, the cellular senescent phenotype, characterized by enlarged cell size, G1/S cell cycle arrest, and increased senescence-associated β-galactosidase (SA-β−Gal) activities were all strongly induced in cells lacking all miR-200s, but importantly not in clones with a residual expression of miR-200 family members. These phenotypes have not been observed previously in cancer epithelial cells with perturbed expression of single miR-200 members. FKO cells also exhibited dysregulated mitochondrial functions, resulting in aberrant metabolic reprogramming, such as augmented oxidative phosphorylation (OXPHOS) with a concurrent downregulation of glycolysis. Using RNA sequencing (RNA-seq) followed by signaling pathway analysis, we comprehensively characterized senescent cancerous epithelial cells with published cellular senescence models in response to different stimuli. We revealed a clear separation of senescent and non-senescent states amongst these cell types. Apart from an enrichment of genes in the EMT signaling pathway, TGF-β and TNF-α pathways were significantly enriched in FKO cells. We also showed that all patients with low tumoural expression of miR-200 family members from the TCGA dataset transcriptomically parallel the EMT subtype cluster of the ACRG cohort. Finally, miR-200 FKO associated-senescence in cancer epithelial cells induced robust stromal cell recruitment, that may contribute to therapy resistance in GC patients with the EMT subtype.

## Results

### miR-200 FKO in GC cells induces significant morphological alteration

**Fig. 1:**
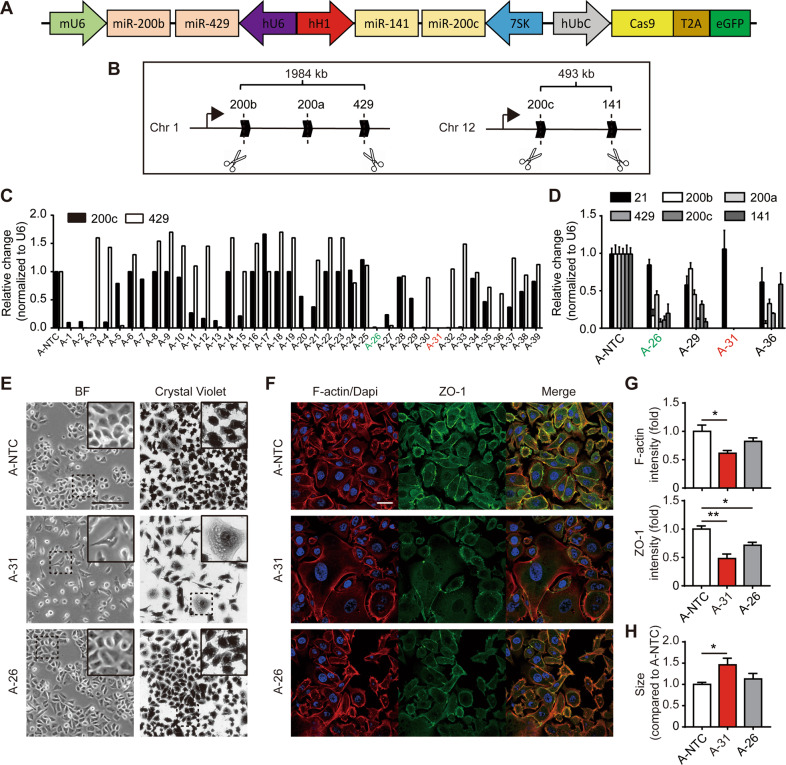
miR-200 FKO induces significant morphological alteration in the human GC cell line, AGS. **A** The multiplex CRISPR/Cas9 in a single lentiviral vector containing four sgRNAs driven by individual promoters. **B** Four sgRNAs indicated by scissors targeting distinct genomic loci of human miR-200 family host genes. **C** qPCR analysis of hsa-mir-200c-3p and miR-429 expression levels as a proxy in 39 single cells derived clonal population of AGS. **D** Further examination of the other miR-200s by qPCR in selected single-cell clones from C with miR-21 as a control. **E** Representative images of bright field (BF) and crystal violet staining illustrates morphological differences of A-NTC cells, the miR-200 FKO clone A-31, and A-26 with residual miR-200s expression from AGS. Scale bar, 100 μM. **F** F-actin (red)/DAPI (blue) and ZO-1 (green) staining of these three AGS clones. Scale bar, 20 μM. **G** Quantification of F-actin and Zo-1 fluorescent intensity from **F**. **H** Flow cytometry analysis of cell size from indicated clones. Data in **D**, **G**, and **H** are presented from triplicate analyses as the mean ± SEM. *p < 0.05, and **p < 0.01.

In order to investigate the effect of global miR-200 family loss in GC cells, we targeted all five members of the miR-200 family located across two different genomic loci using a multiplexed CRISPR/Cas9 system in a single lentiviral vector [10]. A golden gate assembly method facilitated the cloning of four sgRNAs individually targeting hsa-miR-200b-5p, hsa-miR-429, hsa-miR-200c-5p, and hsa-miR-141-3p expression cassettes including an EGFP reporter gene co-expressed with the Cas9 endonuclease (Fig. 1A). As a control, we employed a vector consisting of a poly-T terminator sequence downstream of each independent promoter that was cloned as a Non-target control (NTC) [10]. Two DNA fragments encoding guides against hsa-miR-200b, -a, -429 genes in chromosome 1 and hsa-miR-200c, -141 in chromosome 12 were designed to be deleted in the presence of the four sgRNAs and active Cas9 nuclease, resulting in the simultaneous and efficient knockout of all five “-3p” miR-200 family members (Fig. 1B). After lentiviral transduction and EGFP selection by flow cytometry, we first screened 39 clones for the expression of miR-429 and miR-200c as a proxy for successful locus deletion of miR-200 family members encoded in chromosomes 1 and 12 respectively (Fig. 1C). Further validation of selected four clones was carried out by measuring the expression of the complete panel of miR-200 members deriving clone “A-31” that has undetectable levels of all miR-200s, while the heterozygous clones “A-26”, “A-29”, “A-36” expressed different levels of individual miR-200 family members (Fig. 1D). The host gene deletion in these selected populations by semi-quantitative PCR of designed amplicons was confirmed as shown in Fig. S1A, B. To rule out any possible off-targets induced by the multiplex CRISPR system, we conducted whole-genome sequencing (WGS) analysis of A-31 compared to A-NTC cells. As major off-targets by CRISPR-Cas9 often produce near its target site on the genome [11], we widened the scope and did not find unwanted large DNA fragment deletion and rearrangement in chromosomes 1 and 12 in Table S1. In addition, we measured the expression level of 11 genes within 20 KB flanking the target genome loci in chromosomes 1 and 12 by qPCR. Except *C12orf57* and *PTPN6* with a slight increase in the A-31, other genes exhibited no significant difference in AGS-derived clones (Fig. S1D, E). Thus, we used A-31, the miR-200 FKO clone, and A-26 as a paired control with residual miR-200s expression (partially edited) for further functional studies.

By gross observation, we found that complete loss of the miR-200 family led to marked morphological changes in AGS cells by bright field and crystal violet staining. As shown in Fig. 1E, A-31 cells displayed either enlarged cellular size or irregular, unpolarized geometry. As the rearrangement of the cytoskeleton is the main contributor to cellular polarization, we conducted immunofluorescence to characterize the spatial changes in actin expression in these cells. We found that miR-200 FKO disrupted actin cytoskeletal organization and cell junctions, given that F-actin bundling and ZO-1 expression were markedly diminished compared to those of A-NTC cells (Fig. 1F–H). AGS A-26 cells, with partially deleted miR-200s, exhibited minimal morphological alterations with mild deregulation of polarization (Fig. 1F–H). Given the well-documented role of the miR-200s during EMT, we also examined the gene expression of mesenchymal and epithelial markers by qPCR. The expression level of EMT-specific genes, some of which have been validated as miR-200 targets such as *Zeb1, Snail, MSN*, and *Vimentin* were significantly upregulated in A-31 (Fig. S1C). To confirm the findings of miR-200 FKO in AGS, we also screened and generated FKO and heterozygous clones from MKN28, another epithelial-like GC cell line (Fig. S2A, B), which represented similar phenotypic alterations (Fig. S2C, H). Collectively, the expression of miR-200 members is necessary to maintain the epithelial state and prevent EMT in human GC cells.

### Cells lacking miR-200s display significant G1/S cell cycle arrest

**Fig. 2:**
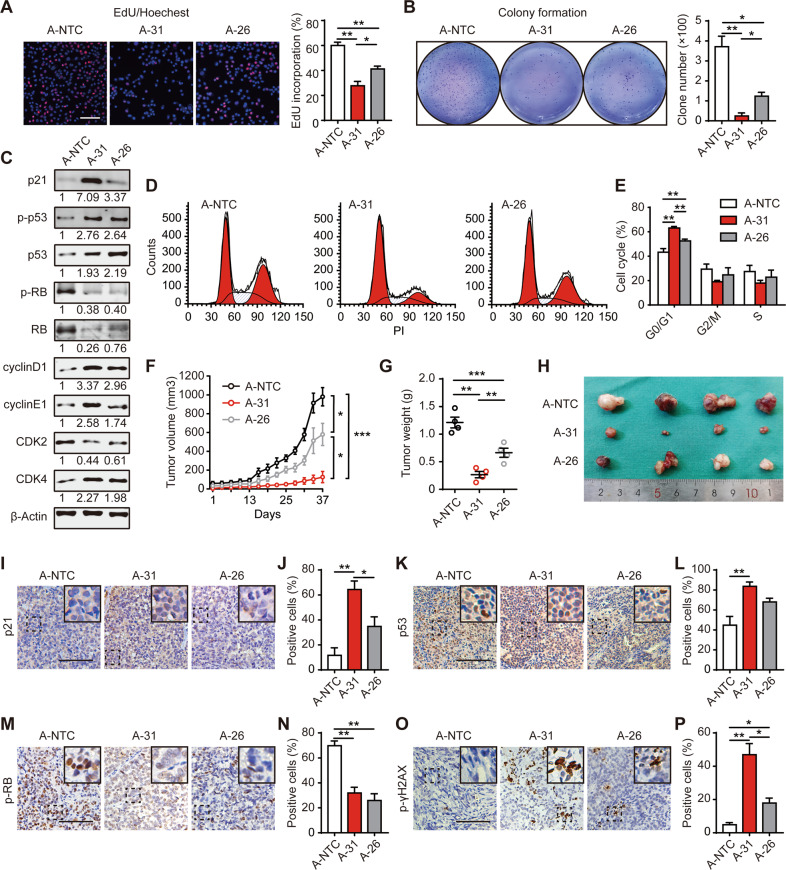
Cells lacking miR-200s display significant G1/S cell cycle arrest. **A** The proliferation of three AGS clones as indicated was measured by EdU. **B** Soft agar assays. Scale bar, 100 μm. **C** Western blot analysis of cell cycle-related proteins with β-Actin as the loading control. **D** Flow cytometry analysis of cell cycle distribution of indicated clones. **E** The percentage of cycle distribution was shown among three clones. **F** Tumor sizes of cell-derived xenografic bearing A-NTC, A-31, and A-26 clonal cells were measured twice weekly (*n* = 4 mice in each group). **G** Tumor weights were measured at the experimental end-point (tumor volume exceeds 1000 mm^3^). **H** Representative images of xenografic tumors inoculated by subcutaneous injection of indicated clones in NSG mice were shown. (**I**, **K**, **M**, **O**) Representative images of xenografic tumors that were subjected to p21, p53, p-RB, and p-γH2AX staining were shown. Scale bar, 200 μm. (**J**, **L**, **N**, **P**) The percentage of positive cells for p21, p53, p-RB, and p-γH2AX was depicted. Data in **A**, **B**, **C**, **E**, **J**, **L**, **N**, and **P** are presented from triplicate analyses as the mean ± SEM. *p < 0.05, **p < 0.01 and ***p < 0.001.

We next assessed the proliferation potential of the A-31 and M-12 clonal populations harboring miR-200 FKO in vitro and in vivo. As depicted in Figs. 2A and S3A, loss of all 5 members significantly inhibited the proliferation rate in the A-31 and M-12 clones as quantified by rates of EdU incorporation when compared to A-26 or M-2 (expressing residual miR-200 expression) and NTC cells. Additionally, anchorage-independent growth of A-31 and M-12 cells in soft agar was also reduced by more than 80% when compared to NTC cells (Figs. 2B, S3B). The growth inhibition of these cells coincided with significantly increased protein expression of cyclin D, CDK4, p53, and its Serine 15 phosphorylated form, as well as the CDK inhibitor p21 CIP (*CDKN1A*, hereafter p21) [12]. In contrast, cells lacking expression of the miR-200 family exhibit hypo-phosphorylated RB (p-RB) and reduced total RB expression (Figs. 2C, S3C). By flow cytometry analysis, we confirmed that A-31 and M-12 cells exhibited significant G1/S cell cycle arrest, whilst A-26 and M-2 show a minimal increase in G1 staging compared to control cells that are in line with their slight elevation of p21 expression (Figs. 2C–E, S3C–E). Consistently, cell-derived xenograft tumors bearing A-31 cells in NOD-scid-gamma (NSG) mice grew much slower than those bearing A-NTC and A-26 cells (Fig. 2F–H). Immunohistochemistry analysis of these xenograft tumors demonstrated that tumor-bearing A-31 cells displayed increased nuclear expression of p21, p53, and p-γH2AX, which facilitates specific DNA repair complexes during DNA damage, whereas p-RB was reduced in A-31 and A-26 (Fig. 2I–P). These observations in vivo were also confirmed in xenografic tumors bearing M-12 cells compared to M-NTC and M-2 cells (Fig. S3F–P). Intriguingly, tumors bearing two heterozygous clones A-26 and M-2 with intermediate proliferative rates showed comparable alteration of p53 and p-RB compared to the FKO counterparts (Figs. 2I–P, S3I–P). We reasoned that this could be due to the equivalent level in response to DNA damage induced by CRISPR-Cas9 [13, 14]. Details of the antibodies used in this study are provided in Supplementary Table S2. Furthermore, apoptosis assays by flow cytometry and the RNA expression of genes encoding the regulation of anti-apoptosis supported the data that reduction in proliferative capacity secondary to miR-200 family loss is not due to an increase in apoptosis (Fig. S4A, B). As the upregulation of p21, the key controller of the cell cycle was consistently present in FKO clones of both lines, we analyzed the potential seed sequence within 3′UTR (untranslated region) showing that p21 is a potential direct target of miR-200s (Fig. S4C). The corresponding 3′UTR luciferase reporter activities of p21 significantly increased approximately three-fold in A-31 cells compared to A-26 or A-NTC cells, but no change in the cells transfected with mutant reporter constructs (Fig. S4D). The qPCR primers sequences of this part are available in Supplementary Table S3. Taken together, miR-200 FKO strongly induced strong G1/S cell cycle arrest in a p21 dependent manner, and p21 could be the direct target of miR-200s in GC cells.

### miR-200 FKO increases lysosomal content and induces an aberrant metabolic phenotype

**Fig. 3:**
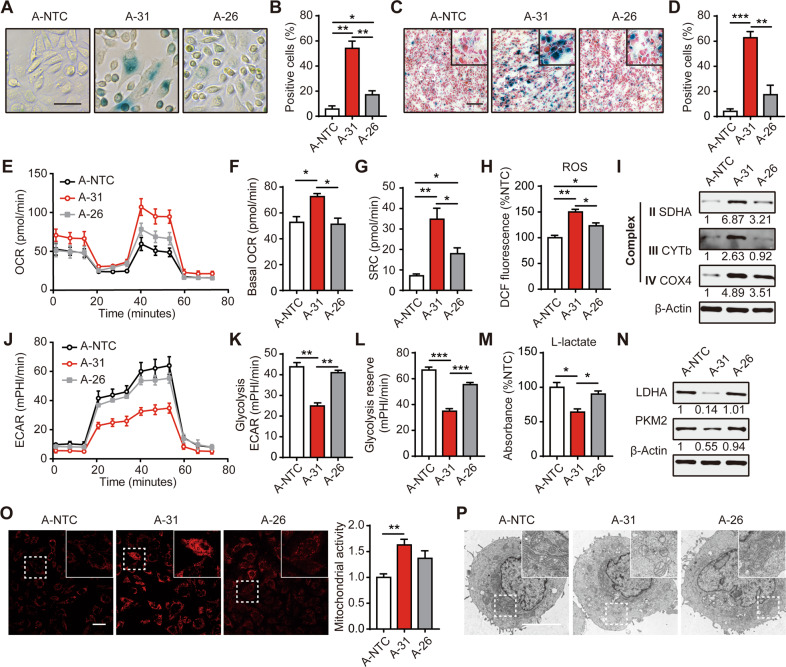
miR-200 FKO increases lysosomal content and induces an aberrant metabolic phenotype. **A** SA-β-Gal staining for A-NTC cells, the miR-200 FKO clone A-31, and A-26 with residual miR-200s expression in vitro. Scale bar, 50 μm. **B** The percentage of cells staining positive for SA-β-Gal from **A**. **C** SA-β-Gal staining for sections from cell-derived xenografic tumor-bearing these three clones in vivo. Scale bar, 100 μm. **D** The percentage of cells staining positive for SA-β-Gal from **C**. **E** Oxygen consumption rate (OCR) was measured using the Seahorse analyzer in these clones with the treatment of oligomycin, FCCP, and a mix of antimycin A and rotenone. **F** Basal OCR and **G** Spare respiratory capacity (SRC) was determined from **E**. **H** Cellular ROS was measured using flow cytometric analysis for DCF stained cells. **I** Western blot analysis of mitochondrial protein complexes in these clones. **J** Extracellular acidification rate (ECAR) was measured using the Seahorse analyzer in these clones with the treatment of glucose, oligomycin, and 2-deoxy-glucose (2-DG). **K** ECAR and **L** Glycolysis reserve was determined from **J**. **M** Lactate production was measured in the culture medium of these cells. **N** Western blot analysis of glycolysis-related proteins in these clones. **O** Staining (left) and quantification of mitochondrial activity (right) of these clones with Mito-Tracker (red). Scale bar, 20 μm. Data represent the mean ± SEM of triplicate independent experiments. *p < 0.05, **p < 0.01 and ***p < 0.001. **P** Representative images of transmission electron microscopy for observing the morphology and structure of mitochondria from these clones. Scale bar, 5 μm.

The morphological alterations and cell cycle arrest observed in miR-200 FKO AGS cells resemble key features of cellular senescence. Therefore, we next assessed the activity of the lysosomal enzyme SA-β−Gal, a commonly used surrogate marker for cellular senescence. Indeed, the SA-β−Gal activity of A-31 and M-12 cells was significantly increased approximately 5-fold compared to NTC cells in vitro and in vivo. In contrast, there was only a moderate increase in the number of SA-β−Gal positive cells in the A-26 and M-2 clones compared to control cells (Figs. 3A–D, S5A–D). We also generated another single lentiviral vector expressing deactivated Cas9 (dCas9) fused to a KRAB repressor domain, in conjunction with sgRNAs targeting the promoter regions to obtain transcriptional repression of miR-200s without inducing double-strand breaks (DSB) (Fig. S5E, Table S4) [15]. We observed a stable 70–90% downregulation of all miR-200 family members for up to 20 days post-transduction in cells with targeted sgRNAs (A-KRAB-200) (Fig. S5F). Consistently, purified A-KRAB-200 cells exhibited increased β-Gal activity compared to A-KRAB-NTC cells with enhanced p21 protein expression (Fig. S5G, H). Although the senescence-associated p21 induction can be suppressed following the transfection of single miR-200 mimics or the combinations by 10–20% as indicated, only overexpression of all 5 miR-200 members in A-31 cells partially rescued the senescent phenotype and led to a greater reduction of p21 expression (Fig. S5I–K). Collectively, these data suggested that miR-200 family members did have collective and summative effects on cellular functions.

Although there is a lack of consensus regarding the metabolic alterations cells undergo during senescence [16], we sought to investigate whether complete miR-200 family loss could lead to metabolic reprogramming. The basal oxygen consumption rate (OCR) and spare respiratory capacity (SRC) were significantly increased in A-31 (Fig. 3E–G). In contrast, the basal extracellular acidification rate (ECAR), which quantifies acid production in cultured media and thus represents the glycolytic rate, was significantly reduced in A-31 cells (Fig. 3J–L). We also found an upregulation of ROS production and reduction of L-lactate in A-31 compared to A-NTC and A-26 cells (Fig. 3H, M). However, A-26 exhibited only a mild increase in FCCP-induced OCR and ROS compared to control cells. By contrast, ECAR, glycolysis reserve and L-lactate production showed no significant difference between A-26 and A-NTC. Western blots confirmed the increased expression of mitochondrial complexes proteins in A-31 (Fig. 3I), whereas the major regulators of glucose metabolism such as PKM2 and LDHA were downregulated in A-31 cells (Fig. 3N). The antibodies are available in Supplementary Table S2. In order to further interrogate the influence of miR-200 FKO on mitochondrial activities, we examined these AGS clones with the Mito-Tracker probe, which stained mitochondria in live cells and reflects mitochondrial membrane potential. We found that Mito-Tracker was significantly concentrated by active mitochondria in miR-200 FKO cells (Fig. 3O). The increased pool of mitochondria in A-31 cells exhibited an abnormal appearance characterized by swelling, crista collapse, and vacuolization, that was associated with their aberrant energy phenotypes (Fig. 3P).

### Cells lacking miR-200s shared senescence-associated gene expression pattern

**Fig. 4:**
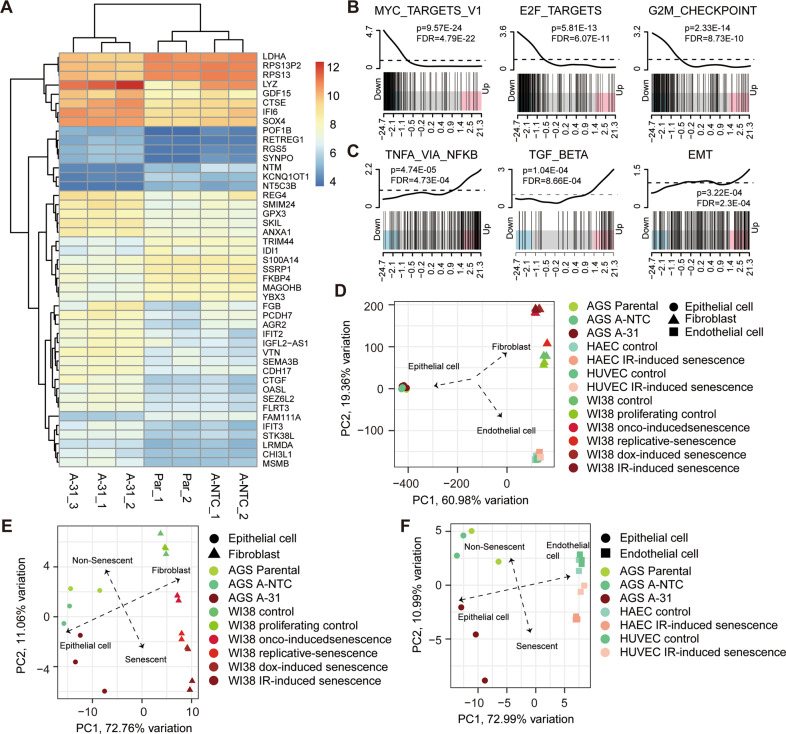
Cells lacking miR-200s shared senescence-associated gene expression patterns. **A** Heatmap displaying the most significantly differentially changed genes at RNA expression levels analyzed by RNA-seq for clones A-31, A-NTC, and the parental AGS cell line (*p* adjusted value *p* < 0.05, absolute logFC>1). Top 3 overrepresented **B** and underrepresented **C** hallmark gene sets from MSigDB as described in Methods in the A-31 cells compared to A-NTC cells identified by CAMERA with *p*-values and FDR as indicated. **D** Principal component analysis (PCA) calculated using the normalized log CPM of all measured transcripts for A-31, A-NTC, and parental AGS cells with GSE130727 (Ref. [17]) showing the cell-type dependent major transcriptional differences. **E**, **F** PCA of the same groups of samples when considering only the 68-gene senescence signature in all models of senescence studied to discriminate the senescent/non-senescent state in the clonal cells.

To further assess the features of miR-200s family loss on mediating a cell senescence program, we interrogated the steady-state transcriptomes of A-31, A-NTC, and parental AGS cells by RNA-Seq. As shown in Fig. 4A, many immune-related genes such as *CTSE* (Cathepsin E) are involved in antigen processing, and *IF16*, *IFIT2*, *IFIT3*, and *OASL* are associated with interferon signaling, were significantly elevated in A-31 cells. In keeping with the metabolic reprogramming observed, genes involved in OXPHOS, glycolysis, and lysosomal function were significantly deregulated, particularly *LDHA*, and *GPX3*. Notably, *GDF15*, a senescent and aging biomarker, and the pro-inflammatory and tissue remodeling genes *CHI3L1* and *SEM3B* were highly upregulated (Fig. 4A). The altered expression profile of these selected genes that play important roles in governing cellular senescence was further validated by qPCR (Fig. S6A–E). We also confirmed that genes linked to proliferation or cell cycles, such as *PCNA*, *c-Myc*, and *p21* were remarkedly altered in A-31 cells (Fig. S6D). Furthermore, gene-set enrichment analysis comparing A-31 with A-NTC or parental AGS control cells demonstrated that the TGF-β, TNF-α, and EMT pathways were most significantly enriched in the absence of the miR-200 family. In contrast, genes involved in Myc targets, G2/M checkpoint, and E2F pathway were strongly underrepresented, in agreement with our findings of an induced cell cycle arrest and low proliferative rate upon global ablation of miR-200s in these cells (Fig. 4B, C).

In order to identify shared transcriptomes of different senescent cell models, we next conducted comparisons using published data from multiple cell types in response to different senescent inducers [17]. Unbiased principal component analysis (PCA) using the normalized log CPM of RNA-seq for all the measured genes in the samples tested revealed that the bulk differences in RNA expression were dependent on the cell type of origin (Fig. 4D). As a shared transcriptome signature with 68 genes was identified from eight diverse models of senescence triggers in human diploid fibroblasts and endothelial cells by various stimuli, we sought to discriminate between the senescent and non-senescent state using a 68-gene “core senescence-associated signature”, as previously described [17]. When using this signature, PCA uncovered a clear separation between A-NTC and parental AGS versus A-31 cell, clustering A-31 with various cell lines that had been inducted into the senescent state (Fig. 4E, F). To experimentally explore our *in silico* analysis, we examined whether the miR-200s have a role in drug-induced senescence in these two GC cell lines. Indeed, low dose of Dox (doxorubicin) and Act D (actinomycin D) as indicated not only induced cellular senescence evidenced by β-Gal activity assay (Fig. S6F, G) but also caused significant suppression of all miR-200 members by 70-90% following 6 days treatment (Fig. S6H, J). Furthermore, p21 gene expression was increased in a dose-dependent manner in these two lines (Fig. S6I, K). Collectively, the gene expression pattern in cells lacking miR-200s shares a common transcriptome to various senescent cells irrespective of cellular origin or the senescent-inducing stimuli.

### The miR-200 family prevents cellular senescence by inhibiting multiple signaling pathways

**Fig. 5:**
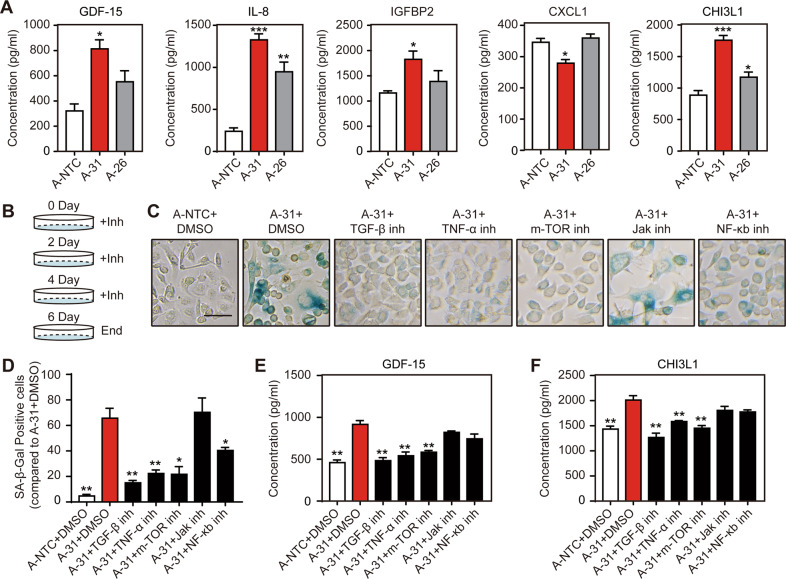
The miR-200 family prevents cellular senescence by inhibiting multiple signaling pathways. **A** Selected secretory proteins identified from RNA-seq analysis were verified using ELISA by harvesting supernatants from 2-day cell culture. **B** The schematic representation for small molecule inhibitors (Inh) was used to investigate the pathways potentially mediating senescence in miR-200 family deficient cells. These pathway inhibitors including TGF-β (RepSox, 1 μM), TNF-α (TNF-α-IN-1, 1 μM), m-TOR (Rapamycin, 10 nM), Jak (CYT387, 1 μM), NF-κb (BAY11-7085, 1 μM) and were renewed in the media every 2 days. **C** SA-β-Gal staining for the miR-200 FKO clone A-31 treated with DMSO vehicle or a variety of drugs inhibiting different signaling pathways for 6 Days. Scale bar, 50 μm. **D** The percentage of cells staining positive for SA-β-Gal from **C**. **E** GDF-15. **F** CHI3L1 in culture media from A-31 cells following the treatment of specific inhibitors for 2 days was measured using ELISA. Data represent the mean ± SEM of triplicate independent experiments. *p < 0.05, **p < 0.01, and ***p < 0.001.

The senescence-associated secretory phenotype (SASP) is a phenotype associated with senescent cells whereby they excrete increased amounts of cytokines, chemokines, and metalloproteinases. We reasoned that the senescent A-31 cells may utilize these secretory proteins to actively regulate the senescent state. We selected three secretory proteins namely GDF-15, CHI3L1, and IL-8 as they were topmost upregulated at the transcriptional level. Further, we also investigated IL-6, IGFBP2, and CXCL1 on the observation that these SASPs ligands are secreted in senescent epithelial cells but not in fibroblasts as previously described [18]. ELISA demonstrated that IL-6 was undetected in the culture media of all three clones, and CXCL1 was decreased in A-31 but unchanged in A-26. The secretion of other selected factors GDF-15, IL-8, IGFBP2, and CHI3L1 was significantly increased in A-31 (with greater magnitude) and A-26 cells compared to that of A-NTC cells. Notably, GDF-15, increased in A-31 media (Fig. 4A), has recently been identified as one of the top “core” SASPs, elevated in many aging-related conditions [18] (Fig. 5A). However, another significantly changed secretory factor, CHI3L1, has not yet been implicated in cellular senescence. The qPCR primers sequences of this part are listed in Supplementary Table S3.

Although we could not detect TGF-β and TNF-α in the supernatant (data not shown), genes involved in TGF-β and TNF-α signaling pathways were most enriched in A-31 cells. We hypothesized that the cultured media from cells lacking all miR-200s established a positive paracrine feedback loop on surrounding cells in order to maintain the senescent state. We sought to use a panel of small molecule inhibitors to mechanistically dissect the contribution of multiple signaling pathways on regulating the senescent state. Out of all the chemical compounds used, we found that the inhibitors targeting TGF-β-receptor 1 (TGFBR1 or ALK5), TNF-α, mTOR, and NF-κB were capable of restraining not only the SA-β-Gal activity induced by miR-200 FKO (Fig. 5B–D), but also the upregulation of p21 expression after only 2 days of treatment (Fig. S7). Interestingly, the JAK inhibitor did affect p21 expression in this setting but did not reduce the number of SA-β−Gal stained senescent cells. Furthermore, the protein expression of GDF-15 and CHI3L1 in cultured media was suppressed following the treatment of specific inhibitors targeting TGF-β, TNF-α, and mTOR pathways (Fig. 5E, F). In contrast, NF-κB inhibition using BAY11-7085 had no impact on these two molecules (Fig. 5E, F), which may be because long-term treatment of this inhibitor also induces G0/G1 phase cell cycle arrest and apoptosis as previously reported [19]. Overall, these data suggest that the loss of the miR-200 family induces senescence which may form a positive paracrine feedback loop involving the key SASPs implicated inactivation of multiple signaling in maintaining a senescent state.

### Genetic deletion of the miR-200 family triggers the recruitment of stromal cells into the tumor microenvironment

**Fig. 6:**
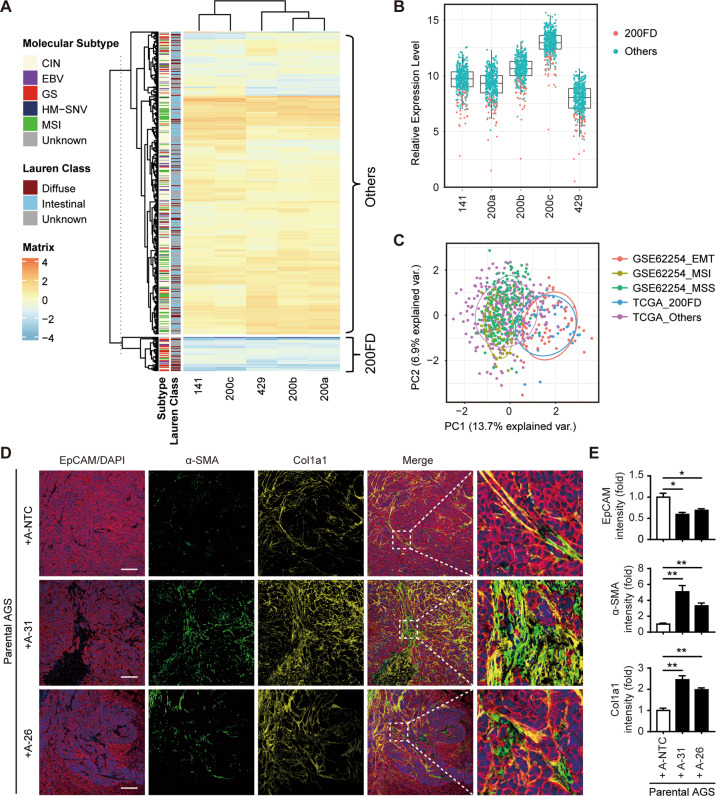
Genetic deletion of the miR-200 family triggers the recruitment of stromal cells into the tumor microenvironment. **A** Expression of all miR-200 family members in TCGA cohort patients (*n* = 430). Patients with all miR-200s lower than 75% GC patients were denoted as “miR-200 family down, 200FD”. **B** Relative abundance of miR-200 family members and those with miR-200s at the bottom 25% in this cohort were shown. Molecular classification of TCGA and Lauren class (left) are depicted. **C** The PCA comparing the transcriptome of the “200FD” subgroup from TCGA with those molecular subtypes identified in ACRG (GSE62254), showing the most similarity between “200FD” and EMT subtype from ACRG. To validate our *in silico* analysis, we inoculated subcutaneously mixed parental AGS and A-NTC, A-31 or A-26 inoculated at a 2:1 ratio of cell number in NSG mice. **D** Immunofluorescent staining for EpCAM (red)/DAPI (blue), α-SMA (green), and Col1a1 (yellow) of sections from xenograft tumors bearing the mixture of cells as indicated. Scale bar, 200 μm. **E** The tumoral purity and the level of stromal recruitment were determined by the quantification of fluorescent intensity for EpCAM, α-SMA, and Col1a1 from **D**. Data represent the mean ± SEM of triplicate independent experiments. *p < 0.05, and **p < 0.01.

To explore the translational potential of our findings, we further analyzed the RNA-seq data of TCGA data sets in patients where the expression of miR-200 family members is lower than in 75% GC patients (Fig. 6A, B). Notably, diffuse type of GC or the molecular subtype of genome stable (GS) were highly enriched in the selected 44 patients (200FD) with expression levels of all miR-200s in the lowest 25% of GC patient tumors. In agreement with the result of the miR-200 FKO AGS and MKN28, signaling pathways such as EMT, Myogenesis, and Fridman senescence were overrepresented when using all mapped coding genes of TCGA, while genes in E2F and G2/M checkpoint pathways controlling cell cycle and proliferation were greatly diminished (Fig. S8A–C). We then performed a comparative analysis of these 44 all-low expressing patients (200FD) with those of different molecular subtypes in another cohort (ACRG, *n* = 300). This cohort was firstly analyzed to characterize the EMT subtype using transcriptome data in GC (GSE 62254). The PCA revealed that those all-low expressing patients from TCGA with enriched senescence-associated genes shared most transcriptome features with those identified as EMT subtype in ACRG (Fig. 6C), which supported the notion that EMT pathway may crosstalk with cellular senescence at the late stage of GC with all low miR-200s expression. As previous studies have reported that the EMT subtype GC is associated with poor patient survival [20], we next conducted a cellular heterogeneity analysis using xCell [21], which integrates the advantages of gene set enrichment with deconvolution approaches, to portray a preliminary EMT associated tumor microenvironment. We found that these 44 patients presented a high score of stromal enrichment in the tumor microenvironment (Fig. S8D).

To experimentally validate our in silico analysis, we inoculated subcutaneously mixed AGS A-31 or A-26 cells with parental AGS cells in NSG mice. Notably, tumor-bearing AGS A-31 mice with a greater growth potential and tumor weight (Fig. S8E–G) demonstrated reduced EpCAM expression and exhibited a significantly increased content of stromal cell infiltration, as labeled with α-SMA (α-smooth muscle actin) or Col1a1 (collagen type I, alpha 1) antibodies compared to A-NTC cells (Fig. 6D, E). Stromal cell enrichment was also moderately increased in tumors bearing the mixture of A-26 and parental cells (Fig. 6D, E). These observations suggested that cancer cells in vivo lacking all miR-200s may comprehensively alter the tumor microenvironment via senescence-associated recruitment of stromal cells.

## Discussion

In addition to its essential role in mediating EMT, previous studies demonstrated that miR-200 family plays not only a tumor suppressor but also a pro-metastasis factor in human cancers [22–24]. Consistent with these studies, we previously showed that overexpression of miR-429, a single miR-200 family member in two GC lines with different histologic subtypes and disease stages, led to pro- or anti-proliferative responses [25]. However, these studies were often conducted by manipulation of a single miR-200 family member. In the light of a close correlation of expression between miR-200 family members during GC initiation and the late stage of the disease [25], whether there exist collective functions of miR-200s working as a complete unit remains unclear. In this study, we employed a multiplex CRISPR system to generate single cell-derived family knock-out clonal cells from two GC lines and revealed a non-canonical function of miR-200s in mediating cellular senescence. Cells lacking all miR-200s underwent strong proliferation inhibition, exhibited multiple key features of cellular senescence, and transcriptomic similarity with EMT subtype of GC. Importantly, the findings of xenografic tumor-bearing FKO clonal cells reflected senescence-associated stroma enriched tumor microenvironment that corroborated the clinical observation [20].

A characteristic cell-cycle arrest, a senescence process often coincides with active metabolic reprogramming. Although it is difficult to speculate whether these GC lines primarily rely on either OXPHOS or glycolysis for their metabolism, both increased OXPHOS and suppressed aerobic glycolysis in AGS cells lacking all miR-200s may be directly associated with the reduction of tumorigenesis. As AGS with residual miR-200a matched the metabolic phenotype of control cells in our study, it seems that the expression of one miR-200 family member is sufficient to maintain energy phenotypes. This is also in line with the data of metabolism-related gene and protein expression analysis comparing A-31, A-26, and A-NTC cells (Fig. 3E–N).

SASPs in the context of cell type and senescence inducers have been extensively studied. The heterogeneity of SASPs limits the usage of a single hallmark marker of senescence [16, 18]. In our study, typical senescence-associated secretory factors previously reported, such as IL-6 and CXCL1, did not alter significantly in A-31 cells, which confirms that some defining SASP components are distinct and vary depending on cell origin and senescence inducer [18]. However, GDF-15, identified as a top “core” SASP and an aging marker in human serum, exhibited a significant increase in transcription and secretion. It has also been reported that GDF-15 is associated with side effects from platinum-based cancer therapy [26], suggesting a close connection between senescence and cancer therapy-related metabolism. Another secretory protein, CHI3L1 was also found to increase at the RNA and protein level in miR-200 FKO cells and has not been reported in any senescence associated pathogenesis. Previous studies showed that this molecule is overexpressed in many human cancers and associated with various malignant behaviors during cancer progressions, such as invasion, angiogenesis, and EMT [27]. In addition, it may be involved in some degenerative diseases including liver fibrosis and coronary artery disease [27]. Considering its ubiquity in the various pathologies, and its abundance in cultured medium, the roles of CHI3L1 in cellular senescence, especially in cancerous epithelial cells and tumor microenvironment, is worthy of future investigation.

Another finding in our study is the influence of senescent epithelial cancerous cells on the tumor microenvironment. Ishimoto etc. previously showed activation of TGF-β in cancer-associated fibroblasts (CAFs) by stimulation with conditioned media from GC cells promoting tumor invasion and malignancy [28], indicating a causal role of cancer epithelial cell-CAFs interaction in the process of tumor development. However, a few studies and our data support the notion that only EMT subtype in the late stage of GC is characterized by stromal enrichment [20]. In our study, the parental AGS cell does not induce fibroblasts to enter into tumoral parenchyma, but the senescent phenotype of AGS with miR-200 FKO does recruit fibroblasts. Our miR-200 FKO AGS cell provides a useful cell model recapitulating the EMT subtypes of GC and the evidence that the initiation of CAF recruitment into the tumor microenvironment may be driven by aging cancerous cells. Considering the limitation of NSG mice with the compromised immune systems in this study, SASP factors in human GC tissue could lead to complex consequences of the disease [29].

Mechanistically, despite that the 3′UTR of p21 as a potential target for miR-200s was identified in this study, how miR-200 family members synergistically control cellular senescence in GC cells is still not clear. Firstly, many altered genes in the signaling pathway of senescence in FKO cells are not recognized as targets of miR-200 family members by seed match. Whether miR-200s mediate these key genes by non-canonical modes, such as different binding sites of miRNA on target gene or gene regulation by non-seed sequence regions of miRNA needs to be further revealed [30–32]. Secondly, a previous study has shown that p53 transcriptionally promotes miR-200s expression and prevents EMT by repressing Zeb1 and Zeb2 expression [33]. Complete loss of miR-200s may lead to an overactivation of p53, which indirectly led to cell cycle arrest in these clonal cells under the enforcement of EMT signaling. Thirdly, miR-200s have also been connected to the regulation of oxidative stress responses and stress-related survival. Xenografic tumors bearing ovarian cancer cells overexpressing miR-141 or miR-200a initiated and grew fast than control counterparts [22]. This was associated with the inhibition of the tumor suppressor gene p38, which activation directly phosphorylates p53, and stabilizes p21 mRNA orchestrating growth arrest [22, 34]. In addition, senescence in these cells may result from an evolutionary process of adaption to lacking all miR-200s and the acquirement of ability to remodel the environment in favor of their survival.

Indeed, as a key effector of EMT, miR-200 family is not the first reported mediator of cellular senescence. Increased activity of Twist-1 and Twist-2 overrides the oncogene-induced senescence in cancer cells [35]. Zeb1 and Snail1, both members of the zinc finger family, facilitate EMT and subvert cell cycle exit from senescence [36, 37]. Moreover, the key regulators of senescence, including Rb and p53 also participate in cross-talk with EMT signaling. Importantly, a number of studies have found that TGF-β ligands, the most important initiators of EMT, are part of the SASP components and actively involved in cellular senescence. These seemingly paradoxical actions of TGF-β indicated a functional link between EMT and senescence. Although the steady reduction of miR-200s often triggers EMT during cancer progression, the intensity of the EMT signaling flux progressively increases during the late stage of cancer until it reaches a threshold that re-activates offset signaling resulting in entry of senescence. This agrees with the findings in A-31 cells with the variable morphology including mesenchymal state and enlarged flatten shape reflecting the different stages driven by escalating EMT signaling. However, whether this EMT-associated senescence is still part of cancer evasion from failsafe programs, such as the strong recruitment of stroma remains to be explored.

Our study demonstrated a non-canonical role of miR-200 family synergistically regulating cellular senescence in GC, mimicking the EMT subtype GC and its tumor microenvironment. We provide new insight of the potential connection and conversion between EMT and senescence mediated by miR-200s. miR-200-SASP-TGF-β axis could be a new target for effective cancer therapy for refractory EMT subtype GC.

## Materials and methods

### Multiplex CRISPR/Cas9 system

Briefly, assembly of custom lentiviral vectors expressing sgRNAs targeting hsa-miR-200b-5p, hsa-miR-429, hsa-miR-200c-5p, and hsa-miR-141-3p were accomplished using Golden Gate cloning into a final lentiviral vector expressing active Cas9 as previously described [10]. Cloning-related primers are available in Supplementary Tables S4 and S5.

Other assays used in this study are described in Supplementary Information.

## Supplementary information

Supplementary Information

Supplementary Information Table S1

Email communication

## Data Availability

The RNA sequencing data for AGS parental, NTC, and FKO clone population has been deposited in figshare. 10.6084/m9.figshare.13699366.v1.
