# Supplementary material for: Complete loss of miR-200 family induces EMT associated cellular senescence in gastric cancer
Source: Oncogene. 2021 Oct 19;41(1):26–36. doi: 10.1038/s41388-021-02067-y (PMC8724006; doi:10.1038/s41388-021-02067-y)
Supplement: Supplementary file 1 — Supplementary Information [file 41388_2021_2067_MOESM1_ESM.docx]

**
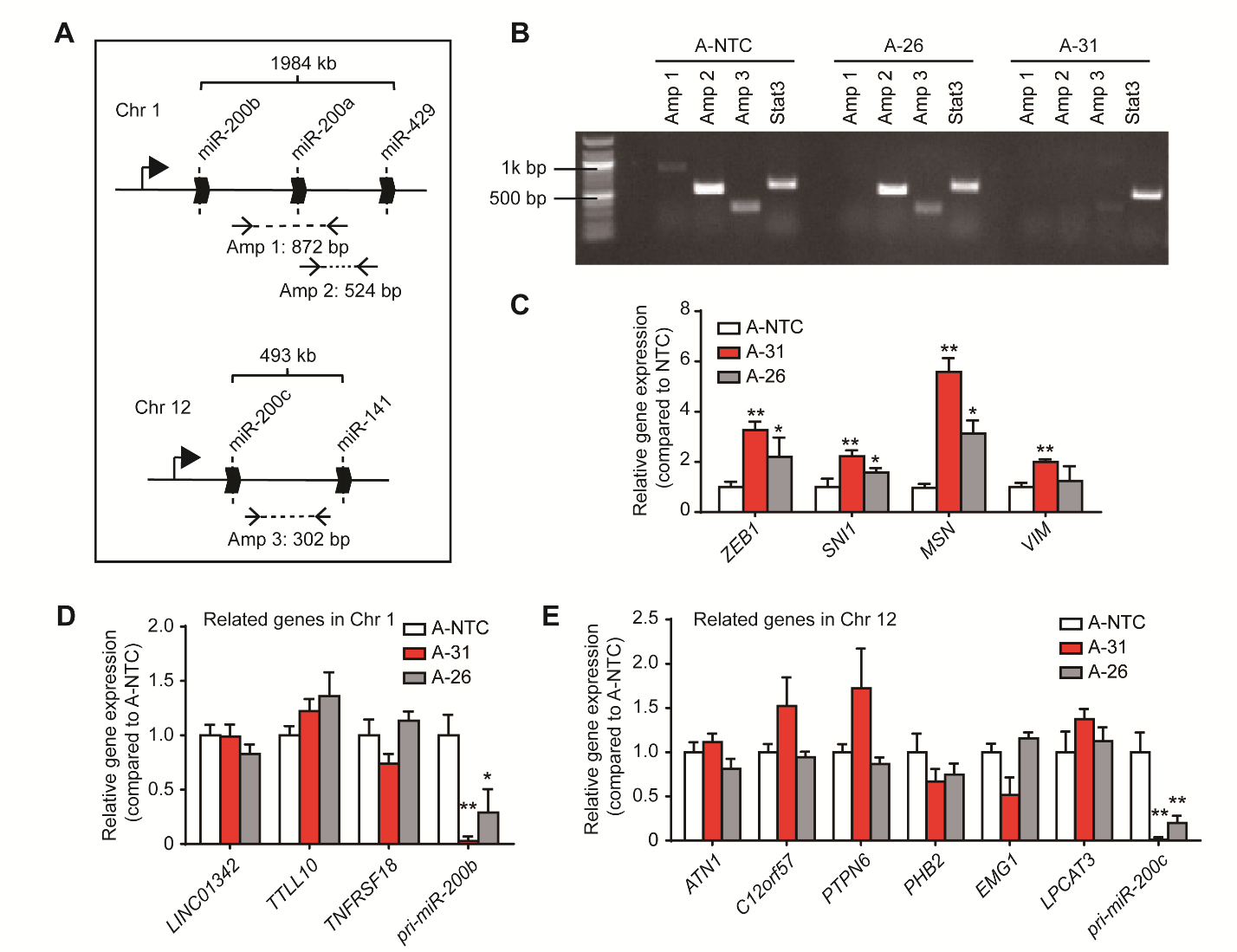
**

**Figure S1: Generation of miR-200 FKO cell in human GC cell line, AGS.**
(A) Designed primers for PCR production detecting genome fragment in human miR-200 family host genes in three clones using semi-quantitative PCR. (B) Gel image showed PCR products from A for three clones with *STAT3* as a control (C) qPCR analysis of genes specifying EMT in these three clonal cells. qPCR analysis of related genes in the proximal region of miR-200 host genes in chromosome 1 (D) or chromosome 12 (E) in A-NTC, A-31 and A-26 clonal cells. Data represent the mean ± SEM of triplicate independent experiments. *p < 0.05 and **p < 0.01.

**
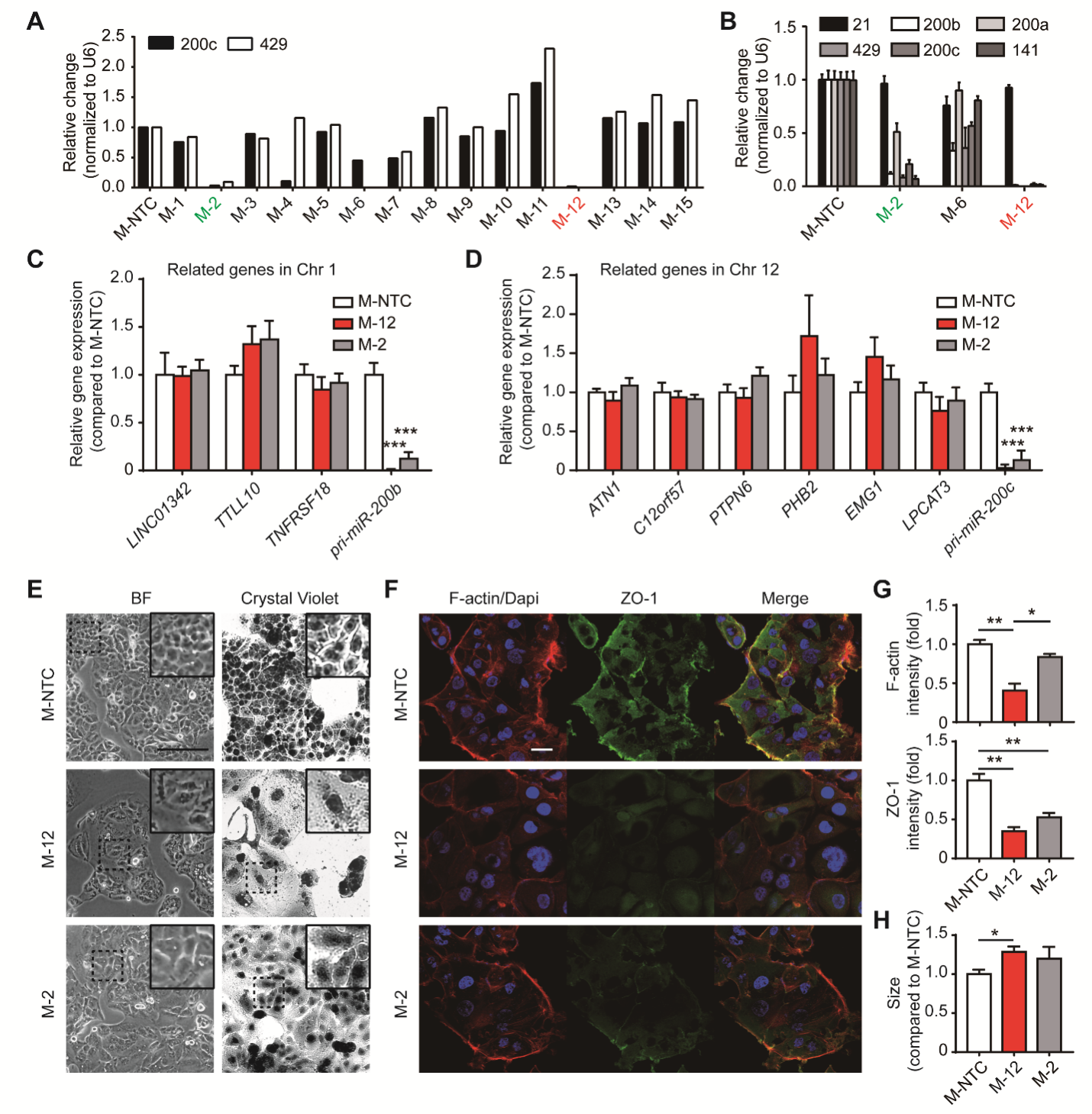
**

**Figure S2: miR-200 FKO induces significant morphological alteration in human GC cell line, MKN28.**

(A) qPCR analysis of hsa-mir-200c-3p and miR-429 expression levels as a proxy in 15 single cell-derived clonal populations of MKN28. (B) Further examination of the other miR-200s by qPCR in selected single cell clones from A with miR-21 as a control. qPCR analysis of related genes in the proximal region of miR-200 host genes in chromosome 1 (C) or chromosome 12 (D) in M-NTC, M-12 and M-2 clonal cells. (E) Representative images of bright filed (BF) and crystal violet staining illustrates the morphological differences of three MKN28 clones. Scale bar, 100 μm. (F) F-actin (red)/DAPI (blue) and ZO-1 (green) staining of these selected clones. Scale bar, 20 μm. (G) Quantification of F-actin and Zo-1 fluorescent intensity from F. (H) Flow cytometry analysis of cell size from indicated clones. Data in B, C, D, G and H are presented from triplicate analyses as the mean ± SEM. *p < 0.05, and **p < 0.01.

**
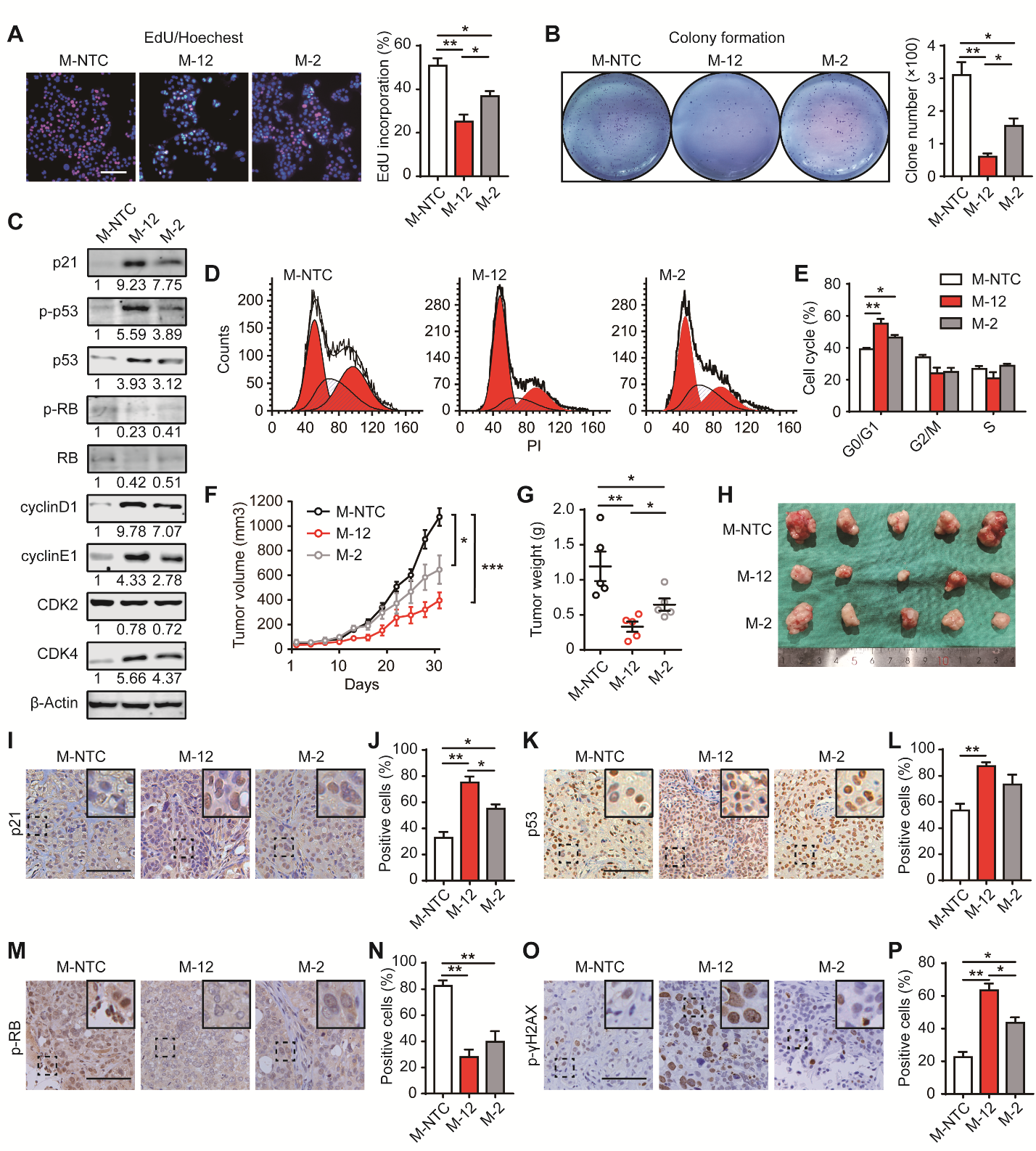
**

**Figure S3: Cells lacking miR-200s display significant G1/S cell cycle arrest in MKN28.**

The proliferation of three MKN28 clones as indicated was measured by EdU (A) and soft agar assays (B). Scale bar, 100 μm. (C) Western blot analysis of cell cycle related proteins with β-Actin as the loading control. (D) Flow cytometry analysis of cell cycle distribution of indicated clones, and (E) the percentage of cycle distribution was shown among three clones. (F) Tumor sizes of cell-derived xenografic bearing M-NTC, M-12 and M-2 clonal cells were measured twice weekly (n=5 mice in each group). (G) Tumor weights were measured at the experimental end-point (tumor volume exceeds 1000 mm^3^). (H) Representative images of xenografic tumors inoculated by subcutaneous injection of indicated clones in NSG mice were shown. (I, K, M, O) Representative images of xenografic tumors that were subjected to p21, p53, p-RB and p-γH2AX staining were shown. Scale bar, 200 μm. (J, L, N, P) The percentage of positive cells for p21, p53, p-RB and p-γH2AX was depicted. Data in A, B, C, E, J, L, N and P are presented from triplicate analyses as the mean ± SEM. *p < 0.05, **p < 0.01 and ***p < 0.001.

**
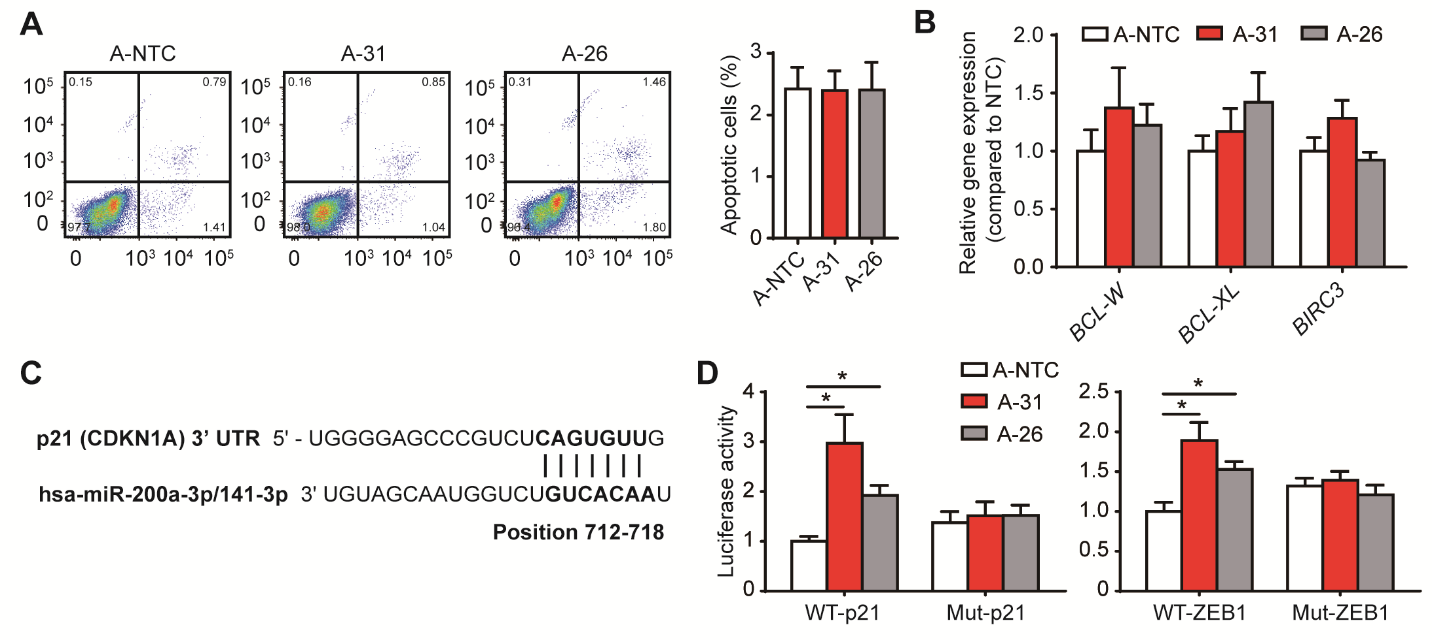
**

**Figure S4: AGS derived clonal cells have no difference in cellular apoptosis; p21 is a direct target of miR-200s in GC cells.**
(A) Apoptosis analysis by flow cytometry in A-NTC, A-31 and A-26 clonal cells. (B) qPCR analysis of apoptosis related genes in three clones. (C) Predicted miR-200 target sites in the 3′UTR of p21 (CDKN1A) by Starbase. (D) Target validation of p21 and ZEB1 as a control was confirmed by luciferase reporter assays in A-NTC, A-31 and A-26 cells transfected with reporter constructs containing WT or mutant seed-matching sequences. WT, wild type; Mut, mutant. Data represent the mean ± SEM of triplicate independent experiments. *p < 0.05.

**
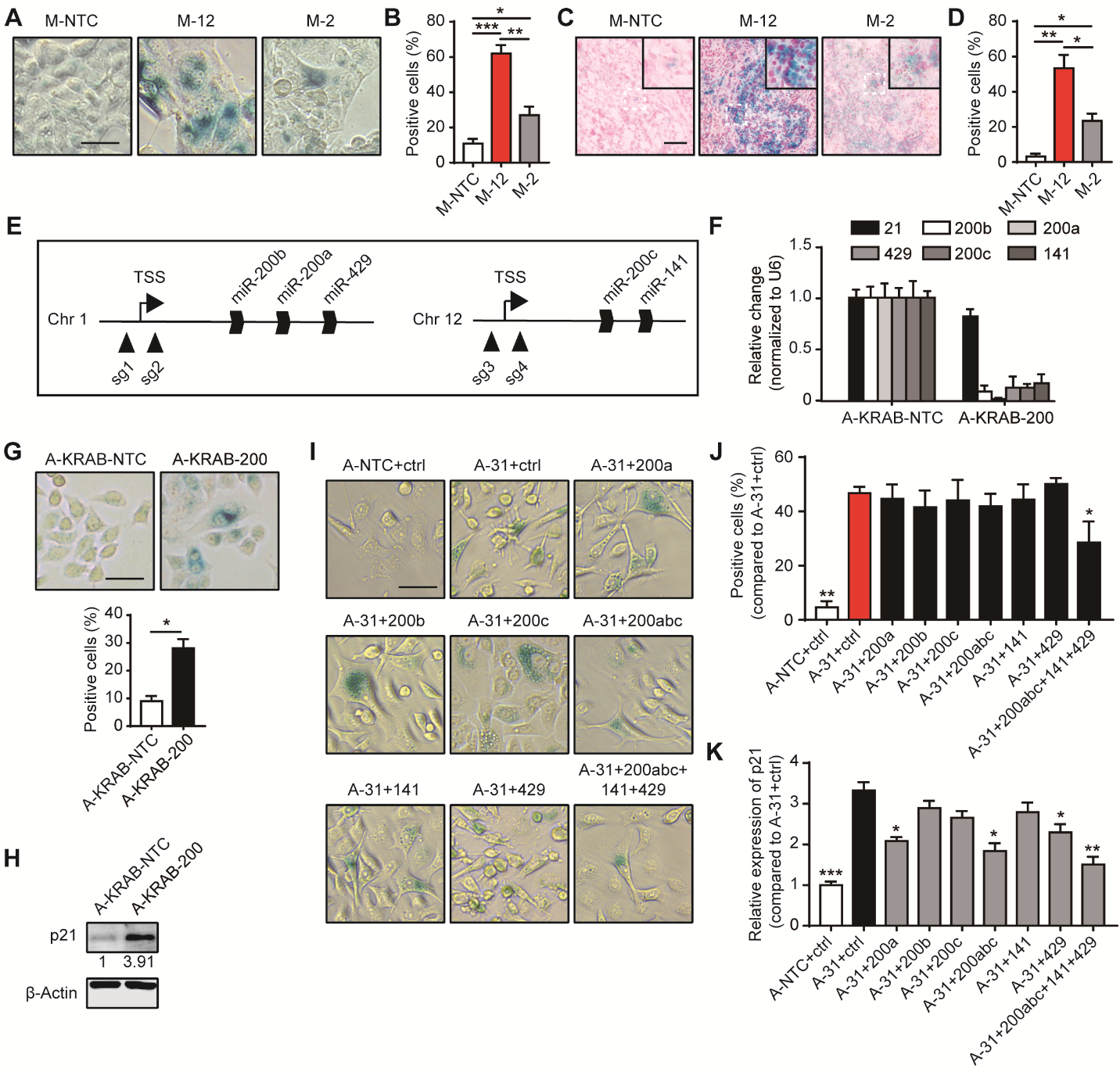
**

**Figure S5:** **Complete loss of miR-200s increases lysosomal content in GC cells**(A) SA-β-Gal staining for M-NTC, M-12, and M-2 cells *in vitro*. Scale bar, 50 μm. (B) The percentage of cells staining positive for SA-β-Gal from A. (C) SA-β-Gal staining for sections from cell derived xenografic tumor bearing these three clones *in vivo*. Scale bar, 100 μm. (D) The percentage of cells staining positive for SA-β-Gal from C. (E) Generation of a single lentiviral vector expressing deactivated Cas9 (dCas9) fused to a KRAB repressor domain (dCas9-KRAB), in conjunction with sgRNAs targeting the regulatory elements of human miR-200s as described in supplementary methods. (F) qPCR validation of miR-200 members in purified A-KRAB-NTC and A-KRAB-200 cells followed by two EGFP-flow sorting rounds. (G) SA-β-Gal staining for A-KRAB-NTC and A-KRAB-200 cells. Scale bar, 50 μm. (H) Western blot analysis of p21 with β-Actin as the loading control. Overexpression of any of miR-200 members or combinations of miR-200 members in A-31 cells by transient transfection of mimetics for 6 days. (I) SA-β-Gal staining for different groups of transfectants. Scale bar, 50 μm. (J) The percentage of cells staining positive for SA-β-Gal from I. (K) qPCR analysis of p21 from these groups of transfectants as indicated. Data represent the mean ± SEM of triplicate independent experiments. *p < 0.05, **p < 0.01 and ***p < 0.001.

**
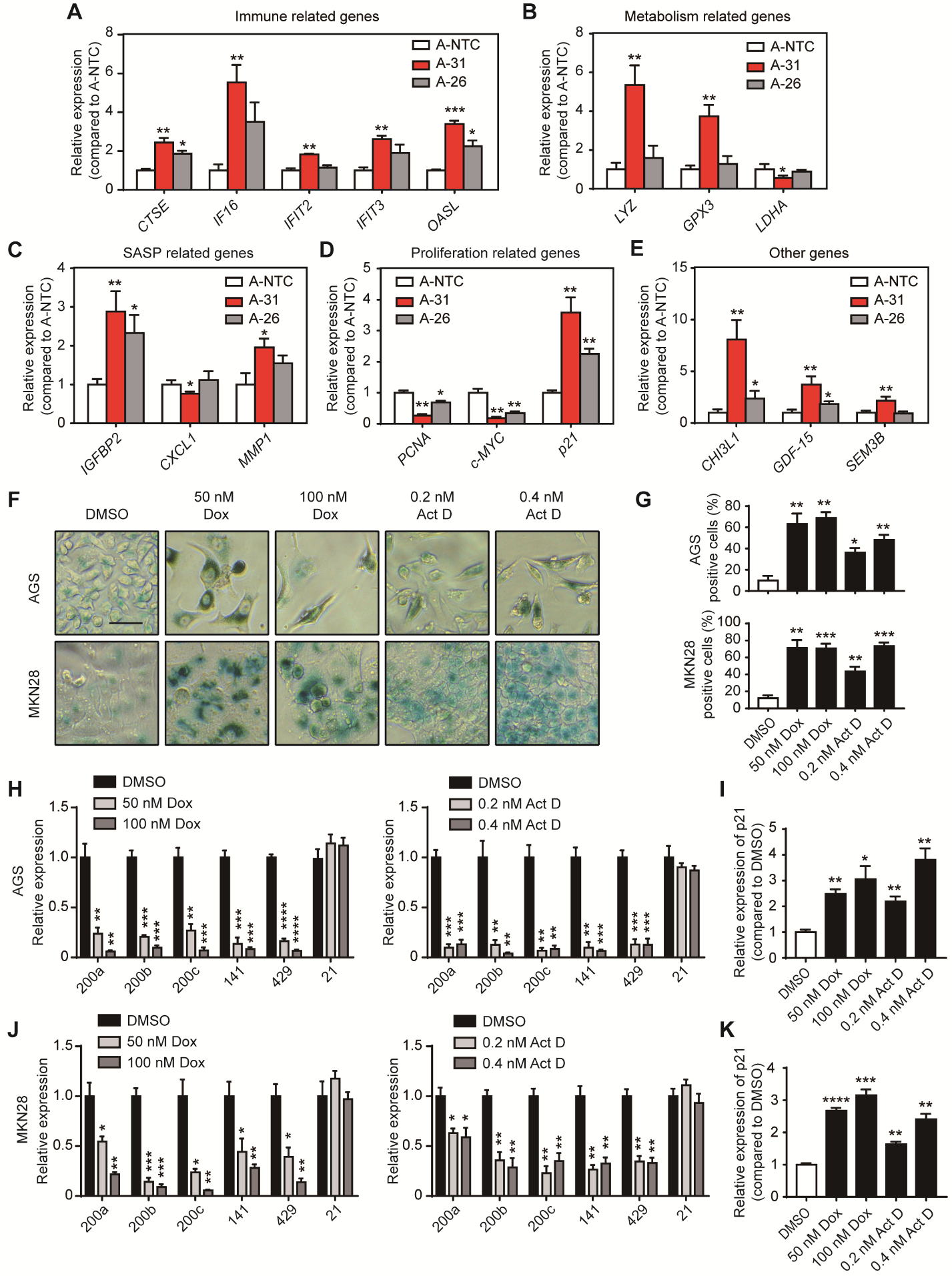
**

**Figure S6: Validation of differentially expressed genes involved in multiple signaling pathways; miR-200s was significantly inhibited in drug-induced cellular senescence.**

qPCR analysis of immune related genes (A), metabolism related genes (B), SASP related genes (C), proliferation related genes (D) or other differentially expressed genes (E) in A-NTC, A-31 and A-26 clonal cells. To induce drug-induced senescence, two GC cell lines were incubated with low dose of Dox (doxorubicin) or Act D (actinomycin D) as indicated concentrations. These drugs were renewed in the media every 3 days. (F) SA-β-Gal staining was conducted in cells following 6 days treatment of DMSO vehicle or compounds. Scale bar, 50 μm. (G) The percentage of positive stained cells for SA-β-Gal assay from F. qPCR validation of miR-200 members (H, J) and p21 (I, K) in drug-induced senescent cells. Data represent the mean ± SEM of triplicate independent experiments. *p < 0.05, **p < 0.01, ***p < 0.001 and ****p < 0.0001.

**
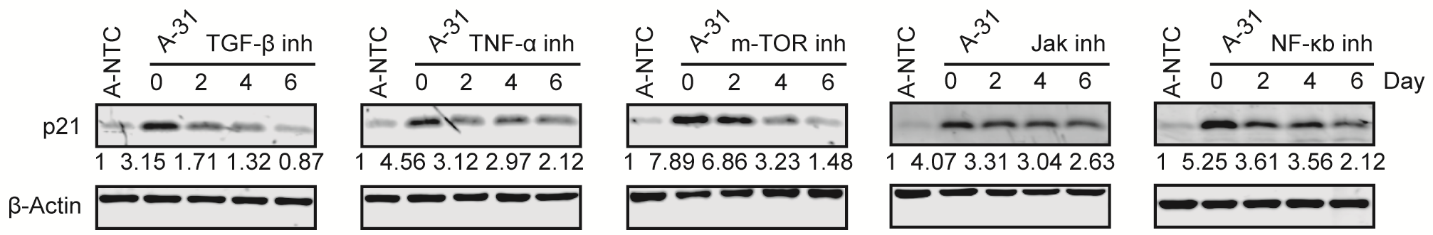
**

**Figure S7: p21 expression of A-31 cells in response to small molecule inhibitors.**

(A) miR-200 FKO A-31 cells were treated with specific pathway inhibitors at the different time points. Cells were then lysed for analysis by Western blotting using specific antibodies to p21, with β-Actin as the loading control.

**
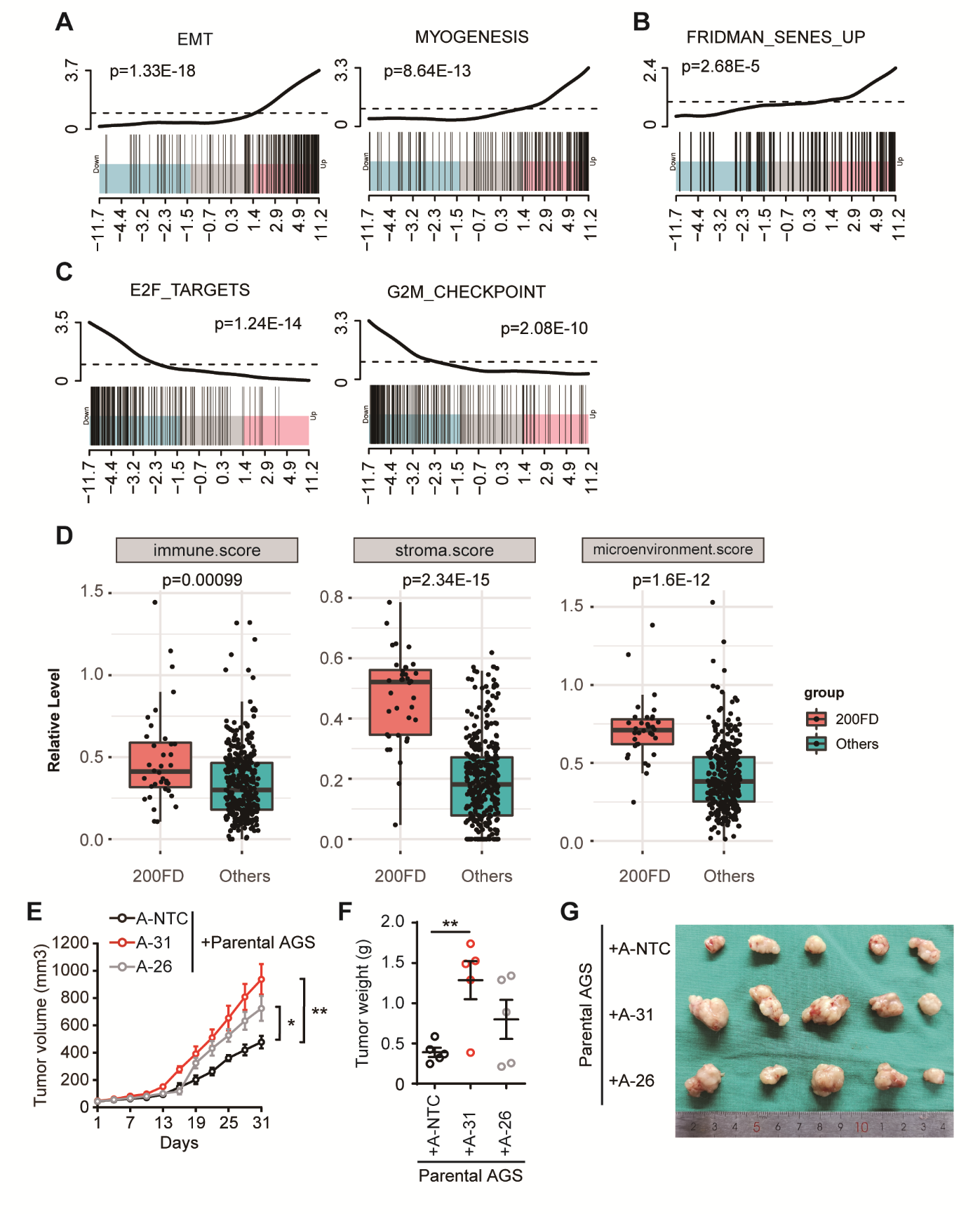
**

**Figure S8: Signaling pathway and cellular heterogeneity analysis using xCELL in all-miR-200-low GC patients.**

(A-C) Top significantly overrepresented or underrepresented signaling pathways in all-miR-200- low GC patients (200FD) in TCGA cohort by CAMERA using Hallmark and curated gene sets from MSigDB as described in Methods. (D) xCell algorithm was applied to estimate the stromal, immune and microenvironment scores of all-miR-200-low GC patients (200FD) in TCGA compared to others based on their transcriptional profiles. Distributions and comparisons of three types of scores among these two groups were shown. (E) Tumor sizes of cell-derived xenograft bearing the mixture of parental AGS with A-NTC, A-31 or A-26 clonal cells were measured twice weekly (n=5 mice in each group). (F) Tumor weights were measured at the experimental end-point (tumor volume exceeds 1000 mm^3^). (G) Representative images of xenograft tumors inoculated by subcutaneous injection of indicated mixed cells in NSG mice were shown. Data represent the mean ± SEM of triplicate independent experiments. *p < 0.05, and **p < 0.01.

**Table S1: Somatic A-31 to A-NTC variants within whole genome sequencing in chromosome 1 and 12 identified by GATK Mutect2 was provided as a separate excel file.**

**Table S2: Antibodies used in this study.**

| **Antibody** | **Application** | **Concentration** | **Company** |
| --- | --- | --- | --- |
| ZO-1 | IF | 1:500 | Proteintech, China |
| p21 | WB/IHC | 1:1000/1:400 | Cell Signaling Technology |
| p-p53 | WB | 1:1000 | Cell Signaling Technology |
| P53 | WB/IHC | 1:1000/1:200 | Cell Signaling Technology |
| p-RB | WB/IHC | 1:1000/1:400 | Cell Signaling Technology |
| RB | WB | 1:1000 | Cell Signaling Technology |
| p- γH2AX | IHC | 1:400 | Cell Signaling Technology |
| cyclinD1 | WB | 1:1000 | Proteintech, China |
| cyclinE1 | WB | 1:1000 | Proteintech, China |
| CDK2 | WB | 1:1000 | Proteintech, China |
| CDK4 | WB | 1:1000 | Proteintech, China |
| Actin | WB | 1:3000 | Proteintech, China |
| SDHA | WB | 1:1000 | ABclonal, China |
| CYTb | WB | 1:1000 | ABclonal, China |
| COX4 | WB | 1:1000 | ABclonal, China |
| LDHA | WB | 1:1000 | Cell Signaling Technology |
| PKM2 | WB | 1:1000 | Cell Signaling Technology |
| Anti-mouse IgG (H+L) | WB | 1:10000 | LI-COR |
| Anti-rabbit IgG (H+L) | WB | 1:10000 | LI-COR |
| EpCAM | IF | 1:1200 | Proteintech, China |
| α-SMA  Col1a1 | IF  IF | 1:2000  1:2000 | Cell Signaling Technology  Cell Signaling Technology |

**Table S3: Primer sequences for qPCR in this study.**

| **Name** | **Forward** | **Reverse** |
| --- | --- | --- |
| ***18S*** | CGGCTACCACATCCAAGGAA | GCTGGAATTACCGCGGCT |
| ***ZEB1*** | GCTTGTGATTTGTGTGACAAGA | AATCGCATGTGTTCAATCAA |
| ***SNI1*** | TGCCCTCAAGATGCACATCCGA | GGGACAGGAGAAGGGCTTCTC |
| ***MSN*** | ACAGTCGCCCCGACGCTAGT | TTGGGCATGGTGGCGGCAAA |
| ***VIM*** | AGGCAAAGCAGGAGTCCACTGA | ATCTGGCGTTCCAGGGACTCAT |
| ***BCL-W*** | CAAGGAGATGGAACCACTGGTG | CCGTATAGAGCTGTGAACTCCG |
| ***LINC01342*** | CCCTGATATTAGCAACCCCAAG | ATTTTCCGAAGACACAAGGGTG |
| ***TTLL10*** | CAGCTCCTACTGCAAAAGCAAG | GTCTCGGCTCTTGACCTCACAC |
| ***TNFRSF18*** | GAGGAGTGCTGTTCCGAGTGG | AGTCGATACACTGGAAGCCAAAAC |
| ***pri-miR-200b*** | CAGCCGTGGCCATCTTACTG | AGGGCTCCGCCGTCATCA |
| ***ATN1*** | GCCACGCAGATCAAACAGGAGC | AAGCCGCGATCCAGGTGTTTGT |
| ***C12orf57*** | GCTTGGTCAAGTCCTACGAAGC | GGCAGAAACAGCGCCTTCAG |
| ***PTPN6*** | TTGACCACAGCCGAGTGATCCT | CTGGCGATGTAGGTCTTAGCGT |
| ***PHB2*** | CACTGAGCAAGAACCCTGGCTA | CATCCTGTAGGTTCAGCACAAGG |
| ***EMG1*** | GATTGAAGTGAATCCCCAGACC | TGTAAAAGTTGAACCATGAGGCC |
| ***LPCAT3*** | TGTGCTTCAGTTCCTCATCCTTC | CGGTGGCAGTGTAATAGTATCCAG |
| ***pri-miR-200c*** | CCCTCGTCTTACCCAGCAGTG | CCTCCATCATTACCCGGCAGT |
| ***p21*** | CTTCCAGCTCCTGTAACATACTGG | CTGTGAAAGACACAGAACAGTACAGG |
| ***PCNA*** | CAAGTAATGTCGATAAAGAGGAGG | GTGTCACCGTTGAAGAGAGTGG |
| ***c-MYC*** | GTGCTCCATGAGGAGACACCG | CAGACTCTGACCTTTTGCCAGG |
| ***BCL-XL*** | ACGAGTTTGAACTGCGGTAC | CTCTGATATGCTGTCCCTGG |
| ***BIRC3*** | GCTTTTGCTGTGATGGTGGACTC | CTTGACGGATGAACTCCTGTCC |
| ***CTSE*** | CAGGTCTGAGAGTTAGGGAAAG | TTCATTGTGAGTCCGACCAG |
| ***IF16*** | CAGCGTCGTCATAGGTAATATTGG | TAACTGGAAGAGTTAGGCCAAGAA |
| ***IFIT2*** | GGAGCAGATTCTGAGGCTTTGC | GGATGAGGCTTCCAGACTCCAA |
| ***IFIT3*** | TGGCCTACATAAAACACCTAGATG | GCTGGATTAACTCTTCAGCTTGC |
| ***OASL*** | TCTGAGGCTGATATGGAAAACC | CTGGATGGTGAAGACGAGAG |
| ***LYZ*** | ACTACAATGCTGGAGACAGAAGC | GCACAAGCTACAGCATCAGCGA |
| ***GPX3*** | GGGGATGTCAATGGAGAGAA | TTCATGGGTTCCCAGAAGAG |
| ***LDHA*** | TGGAGTGGAATGAATGTTG | GATGTGTAGCCTTTGAGTTTG |
| ***IGFBP2*** | GCACATCCCCAACTGTGACAAG | GGGTTCACACACCAGCACTCC |
| ***CXCL1*** | AGCTTGCCTCAATCCTGCATCC | TCCTTCAGGAACAGCCACCAGT |
| ***MMP1*** | AGGTCTCTGAGGGTCAAGCA | CTGGTTGAAAAGCATGAGCA |
| ***CHI3L1*** | CCACAGTCCATAGAATCCTCGG | TGCCTGTCCTTCAGGTACTGCA |
| ***GDF-15*** | GGCCAACCAGAGCTGGGAAG | GCCCGAGAGATACGCAGGTG |
| ***SEM3B*** | GCAAGACCTTTGGCACCTTCAG | CCAACTTGTAGGAAAAGAGGGCG |

**Table S4: Design of oligonucleotides for cloning protospacers into sgRNA expression cassettes.**

| **Oligo under promoter construct** | **sequence** |
| --- | --- |
| **h200b-mU6-O1** | TTGTTTGGCCATCTTACTGGGCAGCAT |
| **h200b-mU6-O2** | AAACATGCTGCCCAGTAAGATGGCCAA |
| **h429-hU6-O1** | CACCGGCCCTCTGTCTAATACTGTC |
| **h429-hU6-O2** | AAACGACAGTATTAGACAGAGGGCC |
| **h200c-7SK-O1** | CCTCGTCGTCTTACCCAGCAGTGTT |
| **h200c-7SK-O2** | AAACAACACTGCTGGGTAAGACGAC |
| **h141-H1-O1** | TCCCATCCAACACTGTACTGGAAGA |
| **h141-H1-O2** | AAACTCTTCCAGTACAGTGTTGGAT |
| **polyT-mU6-O1** | TTGTTTGTTTTTTTTAGCTGACATAA |
| **polyT-mU6-O2** | AAACTTATGTCAGCTAAAAAAAACAA |
| **polyT-7SK-O1** | CCTCGTTTTTTTTAGCTGACATAA |
| **polyT-7SK-O2** | AAACTTATGTCAGCTAAAAAAAAC |
| **polyT-hU6-O1** | CACCGTTTTTTTTAGCTGACATAA |
| **polyT-hU6-O2** | AAACTTATGTCAGCTAAAAAAAAC |
| **polyT-H1-O1** | TCCCATTTTTTTTAGCTGACATAA |
| **polyT-H1-O2** | AAACTTATGTCAGCTAAAAAAAAT |
| **dCas9-KRAB-target 1** | CGGGCCAGTGACGGTGACGCAGG |
| **dCas9-KRAB-target 2** | GGGAGAGTTTCGAGGGCGACGGG |
| **dCas9-KRAB-target 3** | TAAGGCTTGGCCACTCCTCTAGG |
| **dCas9-KRAB-target 4** | GGGCTCTAGGCCGTGGAATCTGG |
| **dCas9-KRAB-NTC 1** | ACGGAGGCTAAGCGTCGCAA |
| **dCas9-KRAB-NTC 2** | CGCTTCCGCGGCCCGTTCAA |
| **dCas9-KRAB-NTC 3** | ATCGTTTCCGCTTAACGGCG |
| **dCas9-KRAB-NTC 4** | GTAGGCGCGCCGCTCTCTAC |

**Table S5: Primer sequences for Semi-PCR.**

| **PCR product** | **Forward** | **Reverse** |
| --- | --- | --- |
| **Amp 1 (872 bp)** | GCCCCTGTGAGCATCTTACC | GCTCGGTCGGGTTAATGAGT |
| **Amp 2 (525 bp)** | TCTGAGGGACAAGGGGTCTC | AGAGGGCCAGGTCTAACCAT |
| **Amp 3 (302bp)** | TCCCTGTGTCAGCAACATCC | CACCAGTTGCTACAGGGGAC |

**Supplementary Materials and Methods**

**Cell culture and transient transfection**

The human GC cell line AGS (American Type Culture Collection, ATCC) were grown in RPMI 1640 (Gibco) supplemented with 10% fetal calf serum (FCS). We have identified the source of cell lines by STR profiling. And the cells were routinely tested for mycoplasma contamination (MycoAlert PLUS Mycoplasma Detection Kit, Lonza). miRNA mimics of human miR-200 family members were purchased from Shanghai GenePharma Company. According to the manufacturer’s instruction, miRNA mimics were transiently transfected into 50%–60% confluent cells with Lipofectamine 3000 (Invitrogen) at a final concentration of 5 nM. For derivation of clonal population expressing different miR-200s, AGS cells were transduced with lentivirus expressing multiplex CRISPR system and grown in culture for 7 days. Single cell clones were then isolated by limiting dilution in 96 well plates. Clones expressing GFP were expanded and genomic DNA and RNA were harvested for analysis.

**Immunoblotting and ELISA**

Cell and tissue lysates were extracted using RIPA lysis buffer containing protease and phosphatase inhibitor cocktail (NCM Biotech, Suzhou, China). Equivalent amounts of protein were electrophoresed on SDS-PAGE gels followed by transferring to PVDF membranes (Millipore, Billeria, MA). Protein bands were visualized and analyzed using the Odyssey Imaging System (LI-COR, USA). The protein expression was quantified by densitometry and normalization to β-actin expression levels. ELISA assays of human GDF-15, IL-8, IGFBP2, CXCL1 and CHI3L1 (Multi Sciences, Hangzhou, China) were performed according to manufacturer’s instruction.

**Immunohistochemistry**

Tissues from xenografts were subjected to deparaffinization, rehydration and heat-induced antigen retrieval to unmask the epitopes, and then incubated with indicated primary antibodies overnight at 4 °C, followed by incubation with the HRP-conjugated secondary detection antibodies (Dako Cytomation) at room temperature for 30 min. The primary antibody was omitted and replaced by a matched isotype control IgG for negative control. The calculation of IHC staining scores was previously described [[1](#_ENREF_1), [2](#_ENREF_2)]. Briefly, the staining results were independently scored by 2 pathologists, and then calculated by multiplying the intensity values (0, no color; 1, weak; 2, moderate; 3, strong), and the proportion values (0, < 5%; 1, 6–25%; 2, 26–50%; 3, 51–75%; and 4, 76–100%). For statistical purposes, the final scores (6-12) were defined as positive staining.

**Immunofluorescence**

Cells were plated on confocal dishes for two days followed by fixation in 4% paraformaldehyde at room temperature for 20 min. After permeabilization with 0.2% Triton X-100 for 5 min, and subsequently incubated in Odyssey Blocking Buffer for 1 h, cells were then incubated overnight with primary antibodies against ZO-1 (1:500, Proteintech, China) followed by incubation with goat anti-Rabbit Secondary Antibody, Alexa Fluor 488 (1: 5000, Invitrogen™, USA), and TRITC-conjugated Phalloidin (1:1000, Sigma-Aldrich, USA). In addition, the multi-staining immunofluorescence of xenografic tumor tissue was performed with primary antibodies (EpCAM, 1:1200; α-SMA, 1:2000; Col1a1, 1:2000) and secondary antibodies (Pano, Beijing, China). At last, immunofluorescence of stained cells was visualized by confocal microscopy (Leica TCS SP8, Germany) and analyzed by Image J software. To label mitochondria, cells were added Mito-tracker™ Red CMXRos (500 nM, Invitrogen™, USA) for 40 min.

**RNA extraction and qPCR**

RNA was isolated using TRI reagent solution (Sigma) followed by the on-column RNeasy mini kit and DNase treatment (Qiagen, Germany). cDNA synthesis was performed using the Transcription First Strand cDNA Synthesis Kit (Roche). qPCR was performed using ABI 7900T PCR System (Applied Biosystems). miRNA using Taqman assays (Applied Biosystems) and gene expression using SYBR Magic were normalized to the expression of U6 or 18S rRNA as described previously [[3](#_ENREF_3)].

**SA-β-Gal assay**

SA-β-Gal activity was measured using a senescence associated β-Galactosidase Staining Kit (Beyotime, Shanghai, China) according to the manufacturer’s instruction. Briefly, cells grown in 6-well plates were rinsed and fixed at room temperature for 15 min followed by incubation with SA-β-gal working solution at 37 °C overnight. For xenografic tumor tissues, frozen tissues were cut for 4 µm by cryotome and mounted onto positively charged glass slides on dry ice. Then sections were fixed and stained as above. SA-β-Gal positive cells were determined from 5 random fields of vision as the percentage of cells staining blue (light or dark blue) with respect to the total amount of cells.

**Flow cytometry and cell growth assays**

Cell proliferation was measured using the EdU assay (RiboBio, Guangzhou, China) and soft agar colony formation assays following manufacturer’s instructions. Colonies were stained with crystal violet and imaged after 20 days of cell culture. Cell cycle was measured with the stains of 1 mg/ml PI and 10 mg/ml RNase A (Sigma). Cell apoptosis was determined with the stains of Annexin V-FITC (Beyotime, Shanghai, China). ROS production was determined with the stains of DCFH-DA (Beyotime, Shanghai, China). The stained cells were then analyzed for cell cycle, apoptosis or ROS by a flow cytometer (BD Biosciences, USA).

**Bioenergetic assays**

Seahorse analyzer (Seahorse Bioscience, USA) was used to measure the mitochondrial and glycolytic activity. Cells were grown in Seahorse miniplates (96 wells) overnight. On the day of assay, each well was confirmed sub-confluent with comparable cell density in the assay plates, after which cells were washed and incubated in Seahorse assay buffer (adjust the pH to 7.4) in a 37 °C non-CO2 incubator for 1 h prior to assay. Pharmaceutical compounds including oligomycin, FCCP, antimycin and rotenone or glucose, oligomycin and 2-DG were reconstituted and made to stressor mix at optimized concentration. The OCR and ECAR were then determined and analyzed by Wave controller software.

**Xenograft tumor model**

6-week-old female NSG mice were used for the animal experiments. All mice were injected into the flank with 5.0 × 10^6^ cells to establish a xenograft model. Each group had 4-6 mice and tumor volumes were measured twice weekly. All mice were sacrificed after tumor volume exceeds 1000 mm^3^. We also inoculated subcutaneously with mixture of AGS A-31 or A-26 cells and parental AGS cells at 1:2 ratio of cell number in NSG mice. Mice that had died from other causes (such as fighting and infection) were excluded. The criteria were pre-established. The mice were allocated to experimental groups and processed using simple randomization. All experiments were approved by the Shanghai General Hospital, Shanghai Jiao Tong University School of Medicine Animal Care and Ethics Committee.

**RNA library preparation and sequencing**

20ng of each total RNA samples was sequenced in-house using Illumina NextSeq550 High output mode and v2.5 chemistry following Illumina protocol 15046563 v04. RNA library preparation and sequencing were performed using 19 bp forward read and 72 bp reverse read with up to 400M reads per run. Fastq files were demultiplexed into each sample based on the 8bp sample index in the forward read. Sample index sequences within 1 hamming distance of the expected sample indexes were included. STAR aligner was used to align the reverse reads to GRCh38. UMI deduplication was performed using the Umi-tools package.

**Gene expression profiling and cellular heterogeneity analysis**

Aligned gene counts were analyzed using the edge R package (Bioconductor release version 3.32.1) in R (version 4.0.3). Raw counts were converted to counts per million (CPM), and trimmed mean of M-values (TMM) was performed to normalize the data for the differential expression analysis. Hallmark gene sets and the curated C2 gene set collection were downloaded from The Molecular Signature Database (MSigDB, version 7.2) and used for gene set testing by the CAMERA method (p value < 0.001 and FDR < 0.01 as cutoff) which is available in the limma R package (version 3.46.0). For cell type enrichment analysis, deconvolution of heterogenous data including TCGA RNA sequencing and ACRG data of microarray was performed and scored by immune, stroma and tumor microenvironment using xCell (version 1.1.0) as previously described [[4](#_ENREF_4)].

**Luciferase reporter assay**

Luciferase reporter assay was performed by transfecting A-NTC, A-31 and A-26 clonal cells with luciferase vectors (empty luciferase vector, luciferase vector containing wild-type target gene 3'-UTR or mutant-type target gene 3'-UTR) for p21 and ZEB1 using Lipofectamine 3000 and performing the Dual-Luciferase Reporter Assay System (Promega, USA) after 24 hours.

**Whole Genome Sequencing**

Genomic DNA was extracted from AGS derived clonal cells using the Qiagen DNeasy kit according to instructions from the manufacturer (Qiagen). Standard instructions from the manufacturer were used for the KAPA DNA Hyper and 150 bp paired-end sequencing reads on the Illumina Novaseq platform with bioinformatic processing and variant annotations. All WGS data on individuals were analyzed according to standardized GATK4 pipeline [[5](#_ENREF_5)] [[6](#_ENREF_6)]. The raw data was aligned to the hg38 human reference genome with the Burrows Wheeler Alignment (v 0.7.17) MEM algorithm [[7](#_ENREF_7)]. Duplication was marked by Picard Markduplicates (v 2.18.0) tool. Base Quality Score Recalibration (BQSR) was performed with GATK tools (v4.1.2.0) before SNP and indel calling was done with HaplotypeCaller on the whole genome. Mutect2 was used to detect somatic mutations. The parameters were based on the GATK Best Practice. CNV was analyzed by CNVkit 0.9.8 as its original tutorial suggests [[8](#_ENREF_8)].

**CRISPRi**

sgRNA confirming to rules including low predicted off-targets and minimal length were designed from a window of 1kb around the TSS of miR-200 host genes. Negative control sequences were selected based on GECKO library. The deactivated Cas9 (DNA 2.0) was fused with two C-terminal SV40 NLSs with the KRAB repressor domain. Using Gibson cloning we cloned these fusion proteins into pLV (Addgene) as previously described [[9](#_ENREF_9)]. sgRNAs were expressed using a lentiviral U6-based expression vector derived from pSico that coexpresses eGFP-T2A-Puro from a CMV promoter. The sgRNA expression plasmids were cloned by inserting annealed oligos into the lentiviral U6-based expression vector that was digested by BstXI and XhoI. We used PCR to amplify the U6 promoter and sequenced the PCR products. Lentivirus and retrovirus were then produced by transfecting HEK293 packaging cell lines with standard 3rd generating packaging vectors. AGS cells with targeted sgRNAs (A-KRAB-200) were generated by transducing cells with a lentivirus expressing dCas9-KRAB from a CMV promoter and the sgRNAs from a U6 promoter. Pure populations of A-KRAB-200 and NTC controls were sorted two times by flow cytometry using a BD FACS for stable EGFP.

**The Cancer Genome Atlas database and the independent cohort of GC**

The mRNA-seq data and miRNA-seq data of GC patients, along with clinical information, were obtained from TCGA data portal (https://portal.gdc.cancer.gov/) in July 2020. The information regarding molecular subtypes of the samples was defined by Liu et al [[10](#_ENREF_10)] and obtained from R package [[11](#_ENREF_11)]. The microarray profiles from 300 gastric tumors and corresponding classification information in the Asian Cancer Research Group (ACRG) were obtained from the Gene Expression Omnibus GSE62254. The sva package was used for removing batch effects between GSE62254 and TCGA-STAD data. PCA analysis was conducted using the PCA tools package (Bioconductor release version 2.0) in R (version 4.0.0).

**Statistical analysis**

All data were shown from at least triplicate independent experiments and presented as mean ± SEM. Two-tailed unpaired t-test were used to determine statistical comparisons, and p-value < 0.05 was considered statistically significant.

**Reference**

1. Liu YD, Yu L, Ying L, Balic J, Gao H, Deng NT *et al*. Toll-like receptor 2 regulates metabolic reprogramming in gastric cancer via superoxide dismutase 2. *International journal of cancer* 2019; 144: 3056-3069.

2. Liu YD, Zhuang XP, Cai DL, Cao C, Gu QS, Liu XN *et al*. Let-7a regulates EV secretion and mitochondrial oxidative phosphorylation by targeting SNAP23 in colorectal cancer. *Journal of experimental & clinical cancer research : CR* 2021; 40: 31.

3. Yu L, Wu D, Gao H, Balic JJ, Tsykin A, Han T-S *et al*. Clinical Utility of a STAT3-Regulated miRNA-200 Family Signature with Prognostic Potential in Early Gastric Cancer. *Clinical Cancer Research* 2018; 24: 1459-1472.

4. Aran D, Hu Z, Butte AJ. xCell: digitally portraying the tissue cellular heterogeneity landscape2017; 18: 220.

5. McKenna A, Hanna M, Banks E, Sivachenko A, Cibulskis K, Kernytsky A *et al*. The Genome Analysis Toolkit: a MapReduce framework for analyzing next-generation DNA sequencing data. *Genome Res* 2010; 20: 1297-1303.

6. DePristo MA, Banks E, Poplin R, Garimella KV, Maguire JR, Hartl C *et al*. A framework for variation discovery and genotyping using next-generation DNA sequencing data. *Nat Genet* 2011; 43: 491-498.

7. Li H, Durbin R. Fast and accurate long-read alignment with Burrows-Wheeler transform. *Bioinformatics* 2010; 26: 589-595.

8. Talevich E, Shain AH, Botton T, Bastian BC. CNVkit: Genome-Wide Copy Number Detection and Visualization from Targeted DNA Sequencing. *PLoS Comput Biol* 2016; 12: e1004873.

9. Thakore PI, D'Ippolito AM, Song L, Safi A, Shivakumar NK, Kabadi AM *et al*. Highly specific epigenome editing by CRISPR-Cas9 repressors for silencing of distal regulatory elements. *Nat Methods* 2015; 12: 1143-1149.

10. Liu Y, Sethi NS, Hinoue T, Schneider BG, Cherniack AD, Sanchez-Vega F *et al*. Comparative Molecular Analysis of Gastrointestinal Adenocarcinomas. *Cancer cell* 2018; 33: 721-735.e728.

11. Colaprico A, Silva TC, Olsen C, Garofano L. TCGAbiolinks: an R/Bioconductor package for integrative analysis of TCGA data2016; 44: e71.
