# Supplementary figures and images for: Complete loss of miR-200 family induces EMT associated cellular senescence in gastric cancer
Source: Oncogene. 2021 Oct 19;41(1):26–36. doi: 10.1038/s41388-021-02067-y (PMC8724006; doi:10.1038/s41388-021-02067-y)

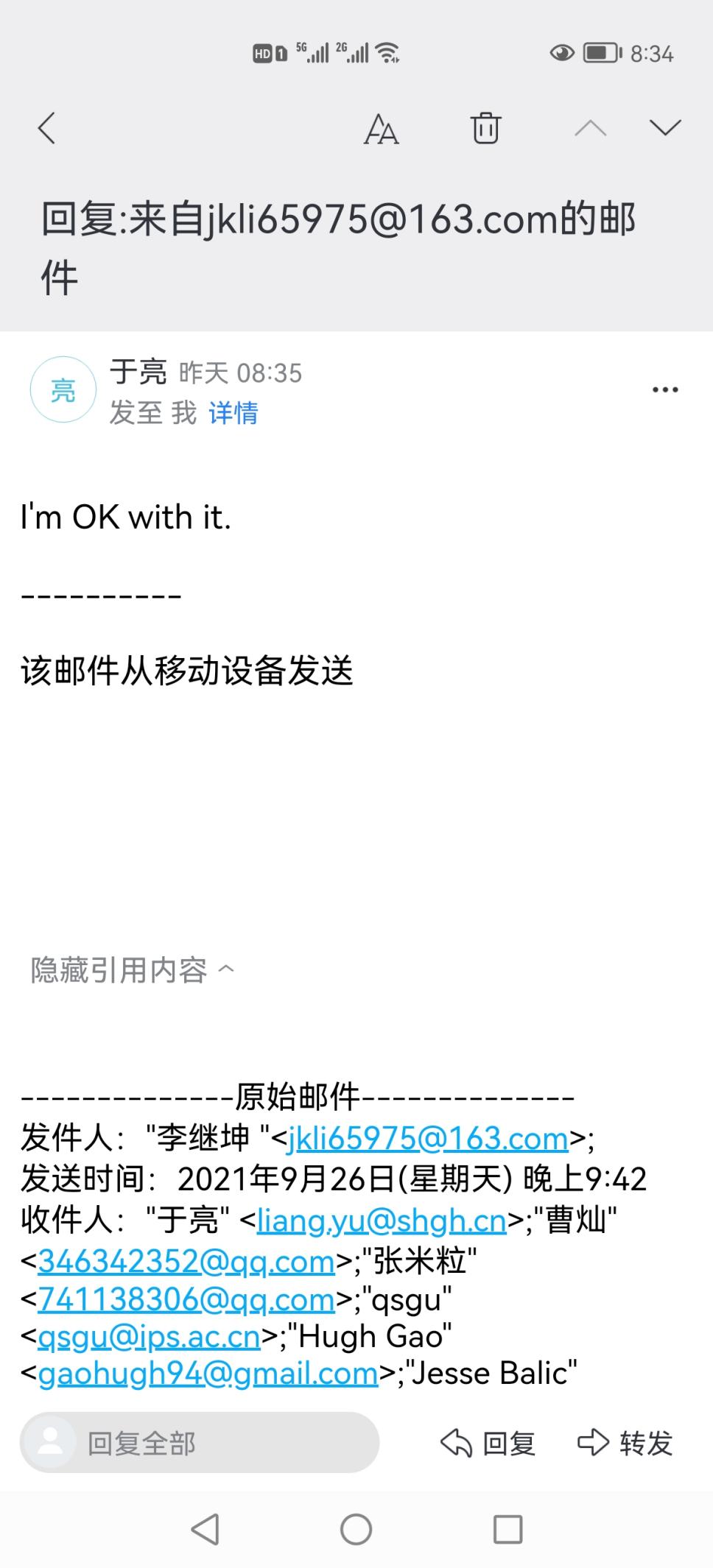


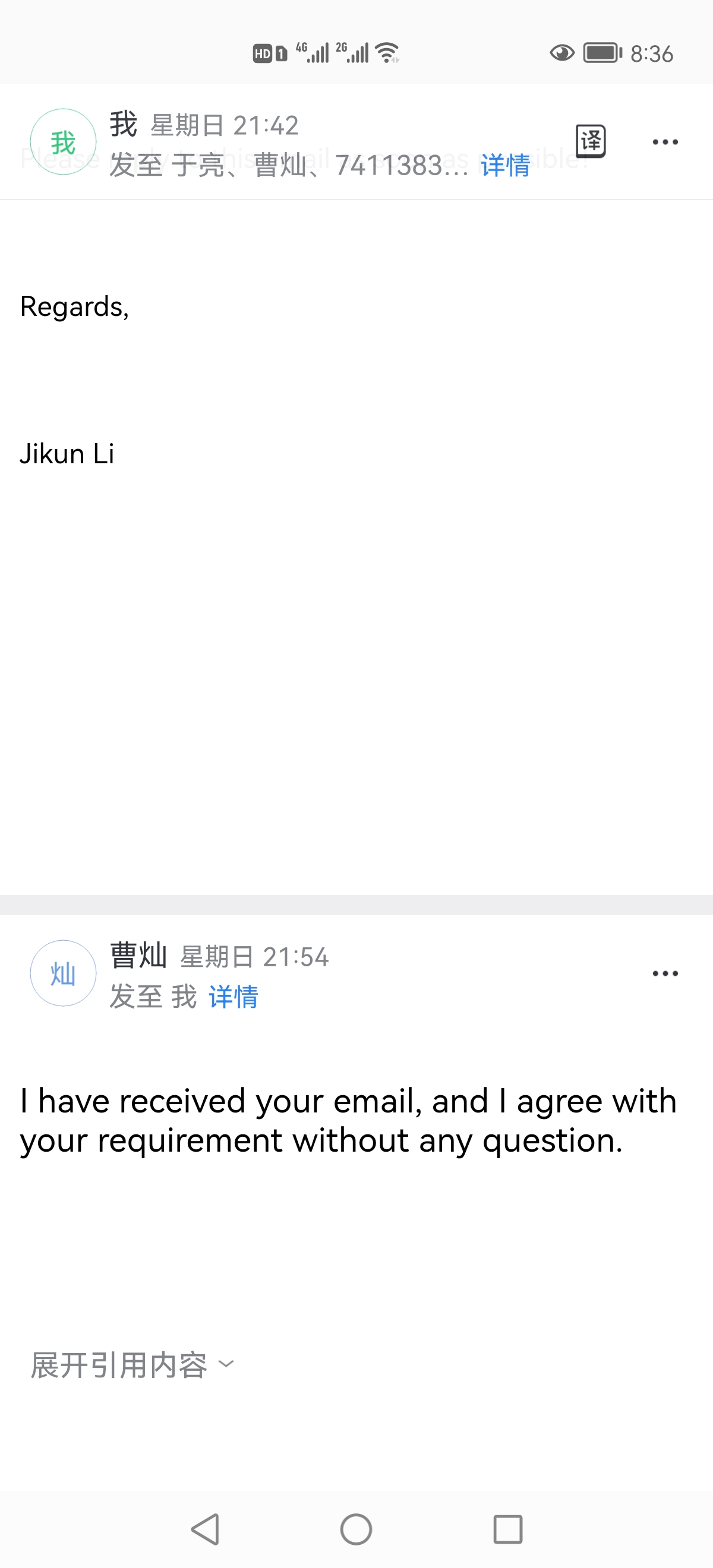

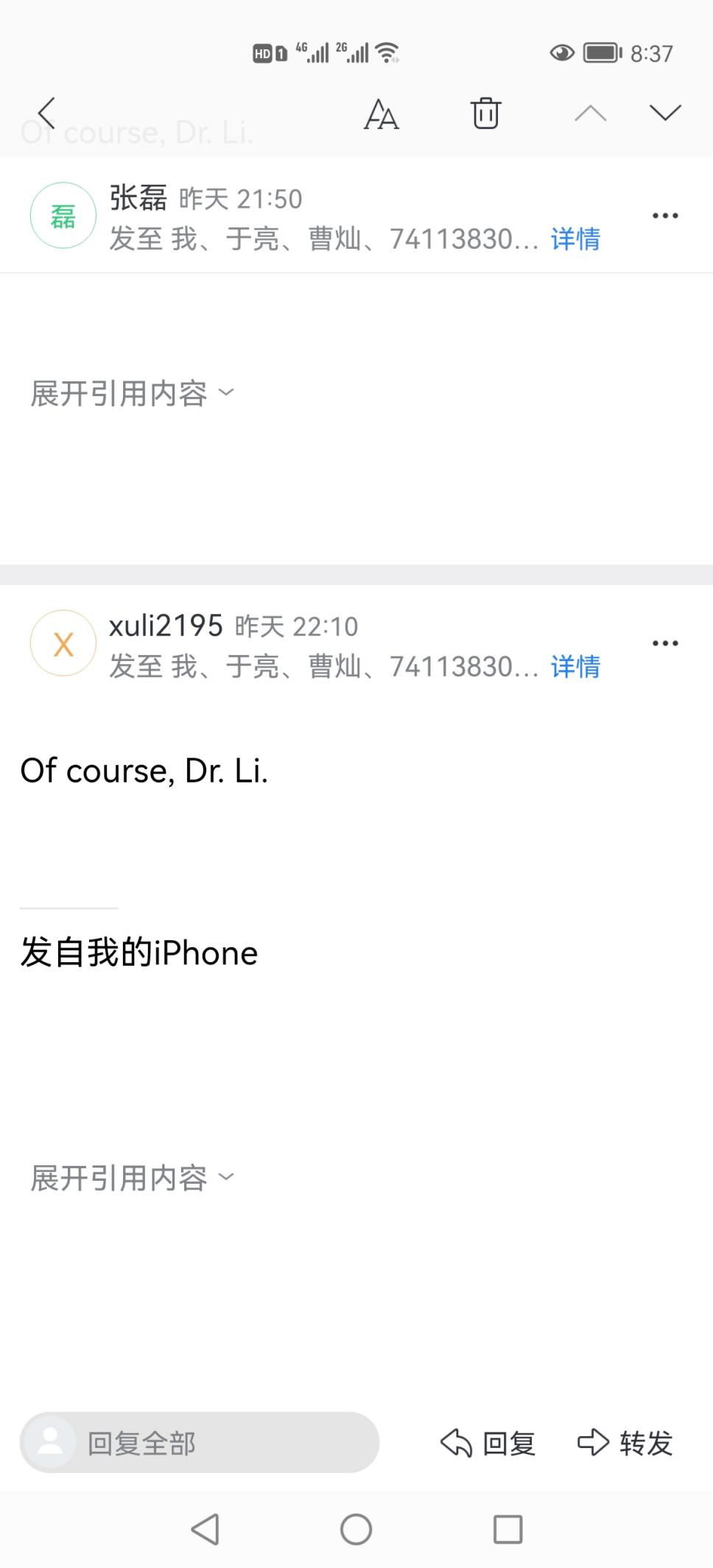

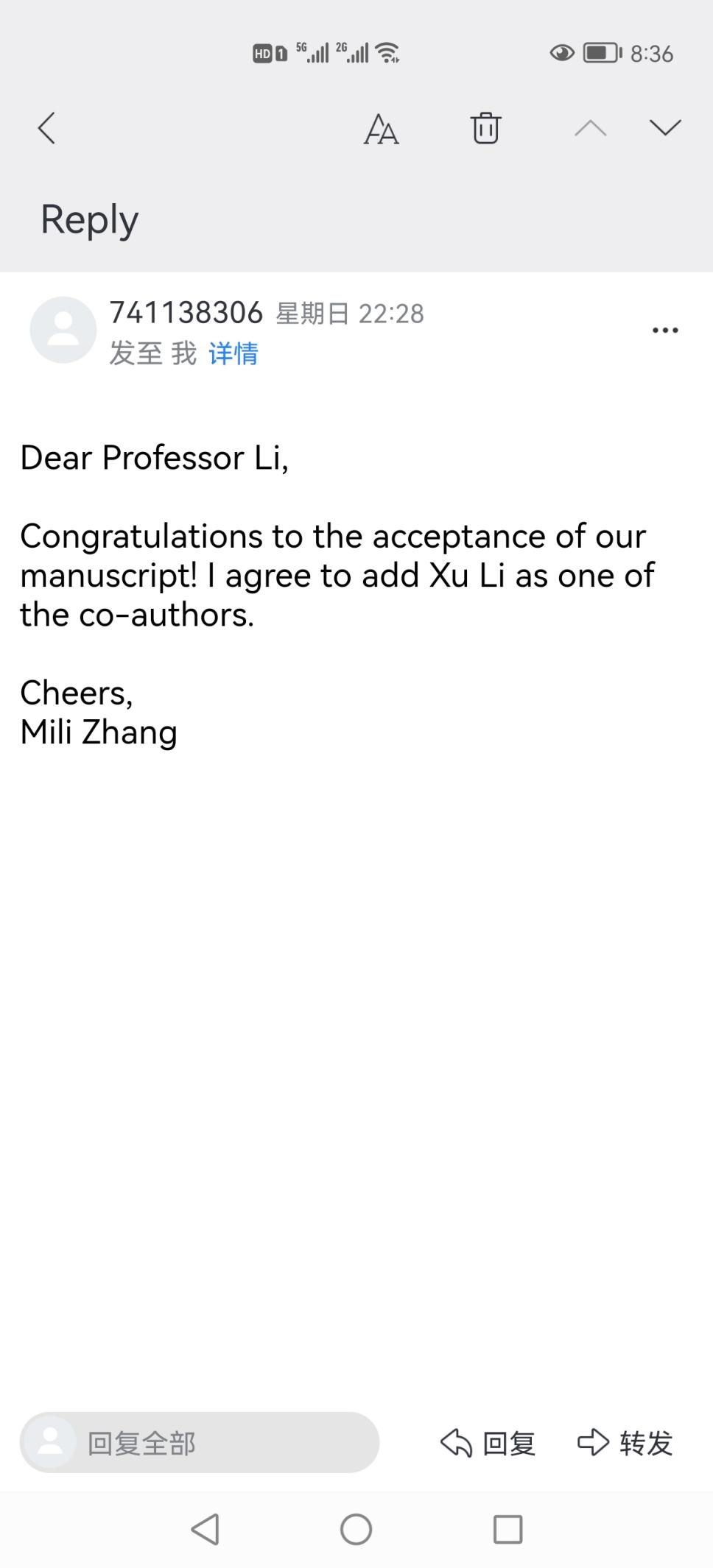

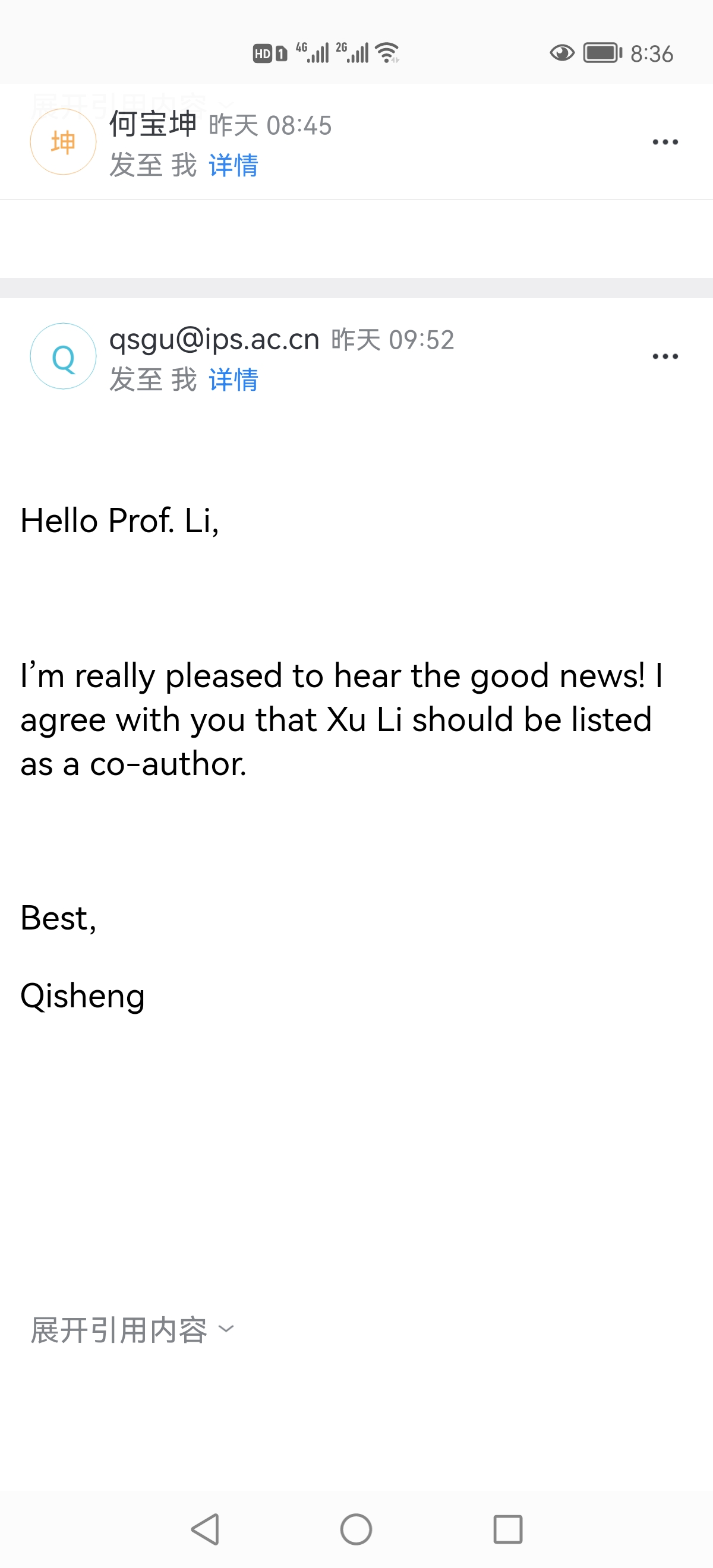

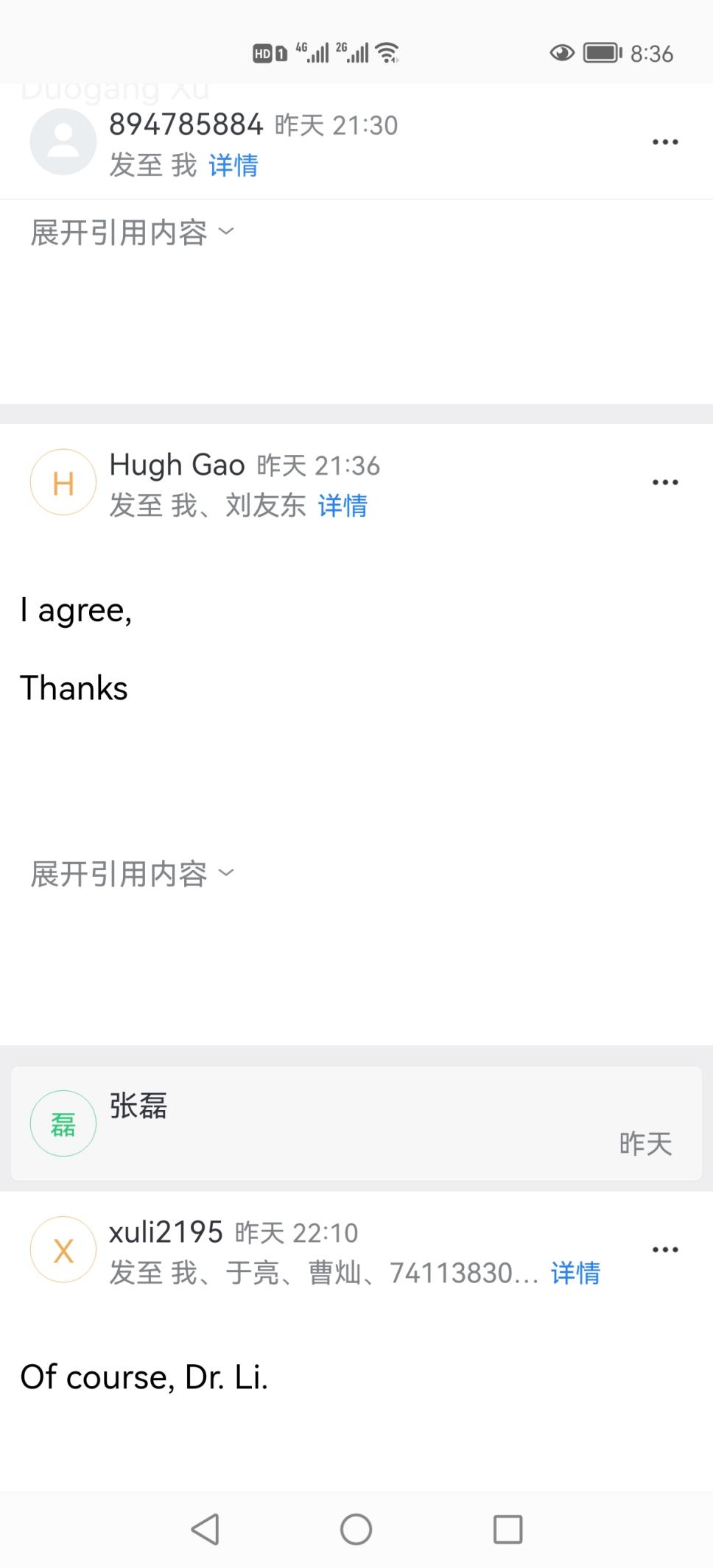

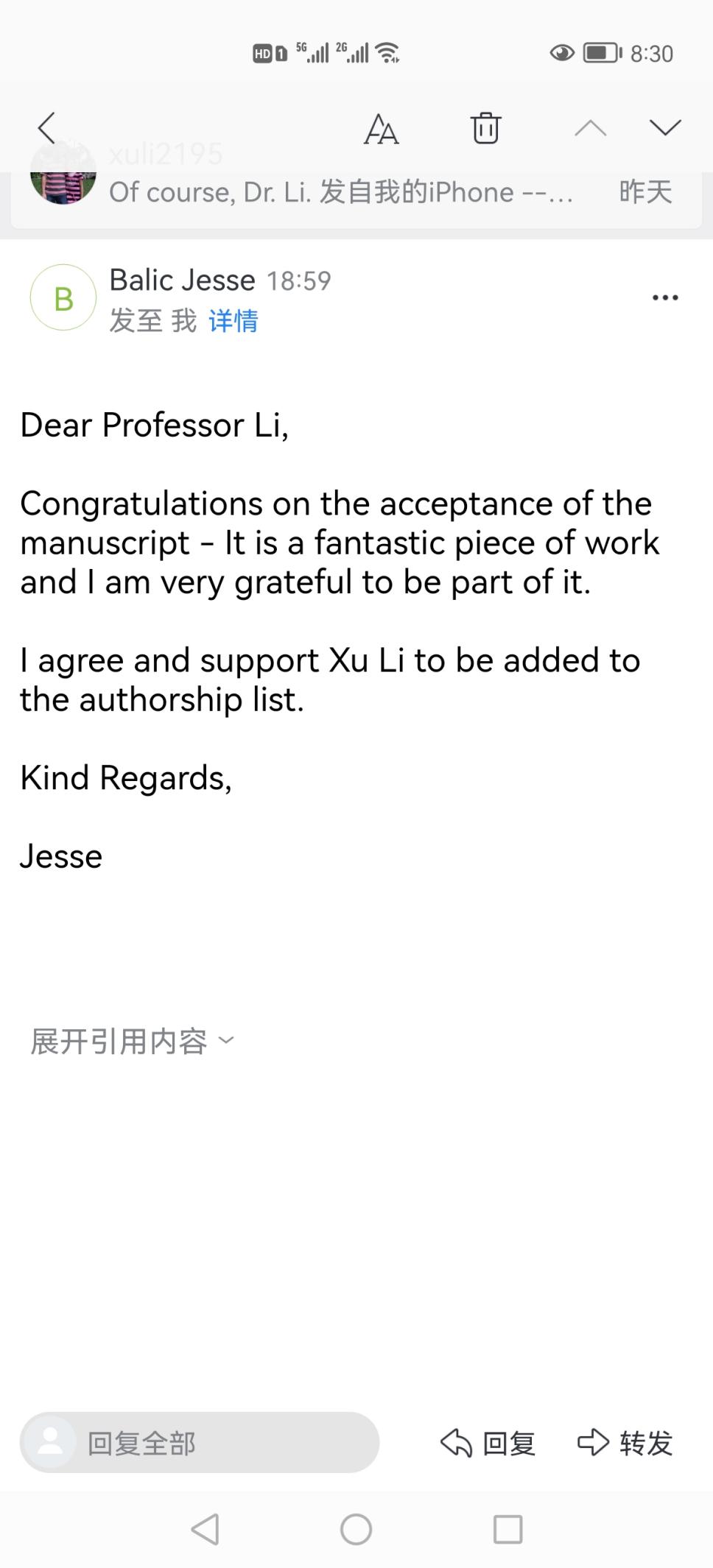

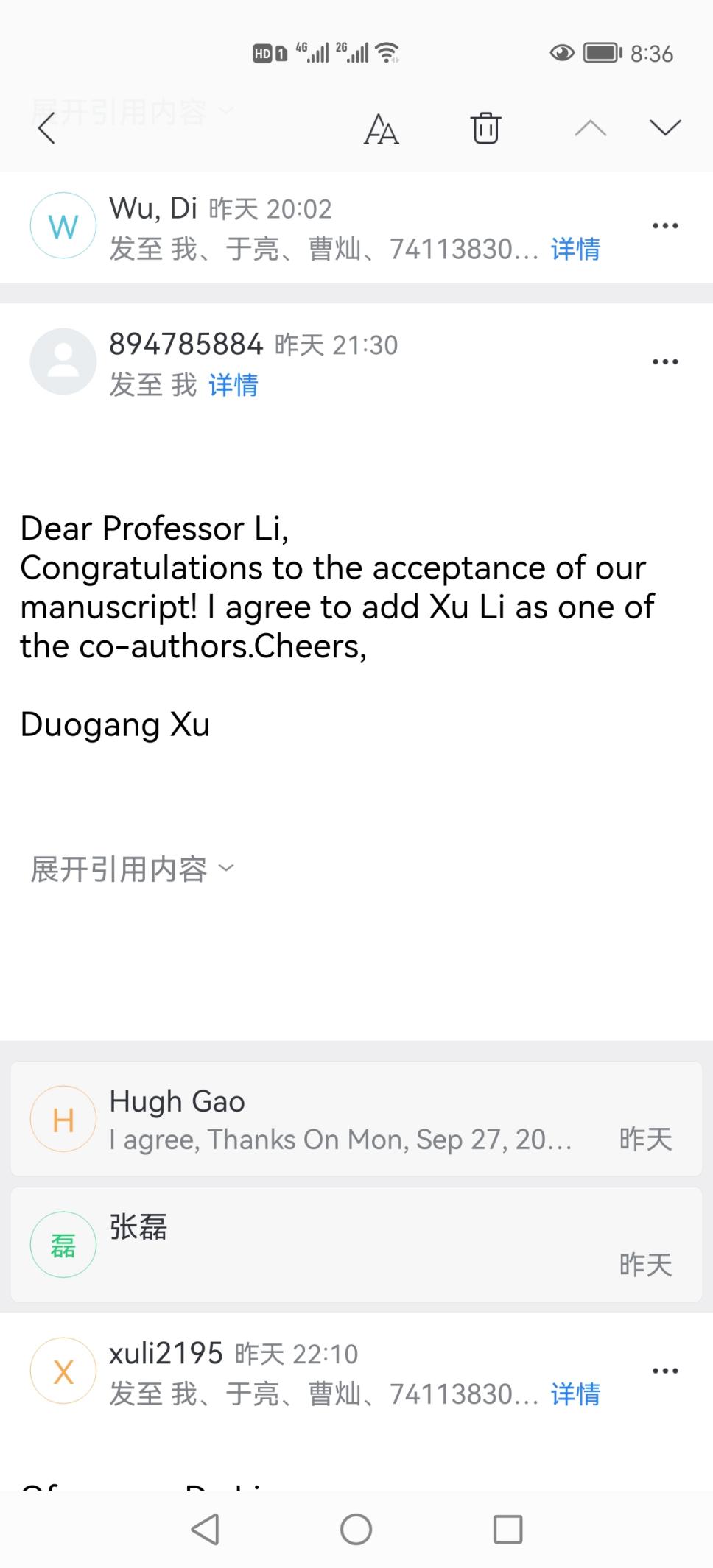

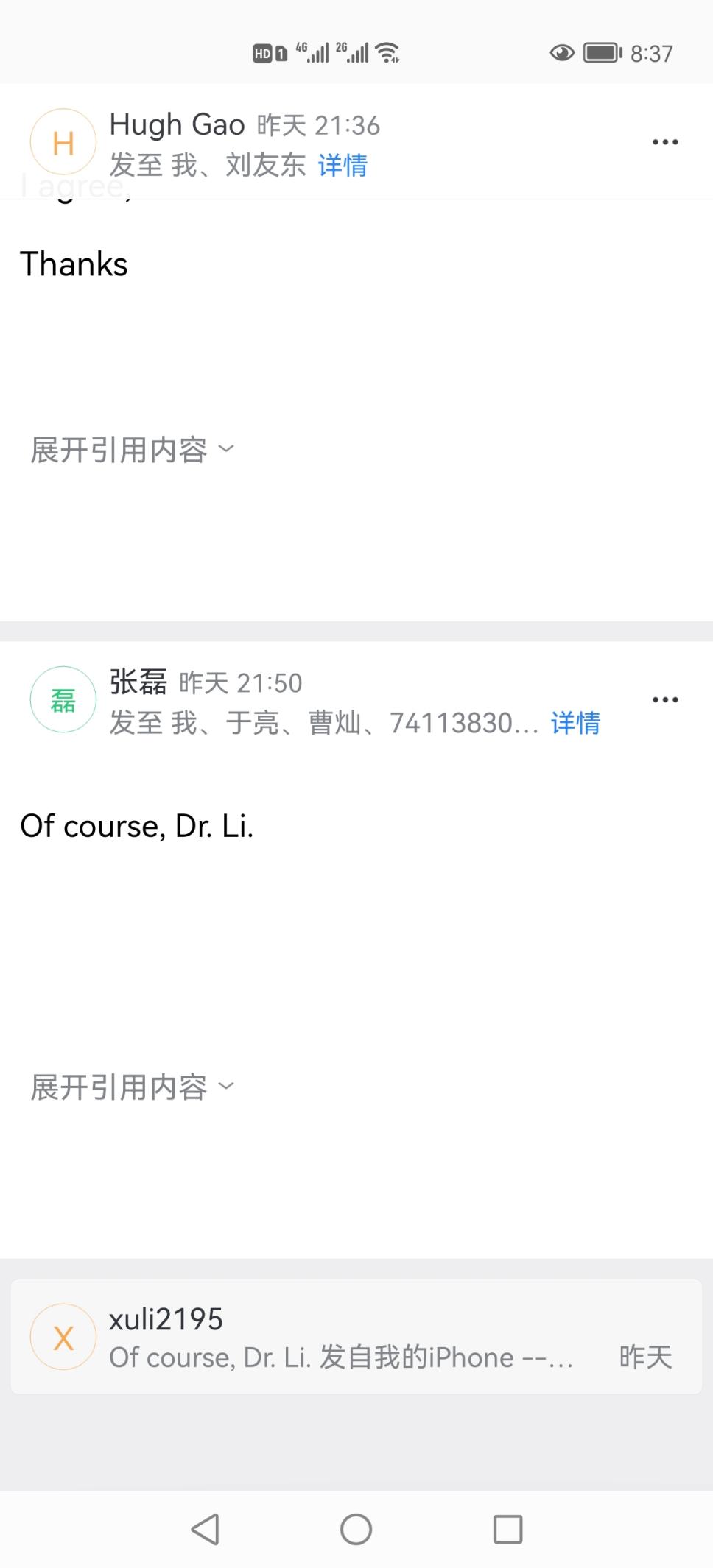

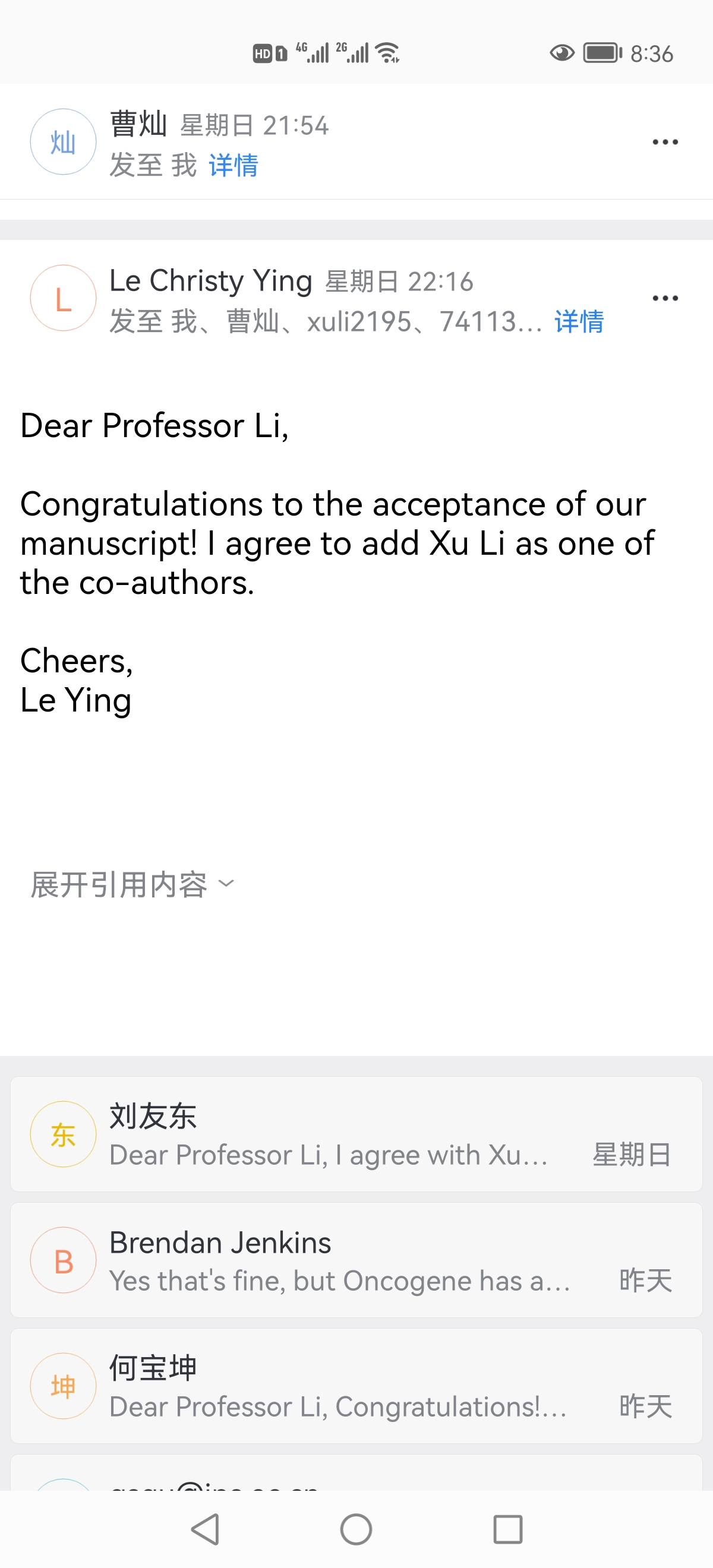

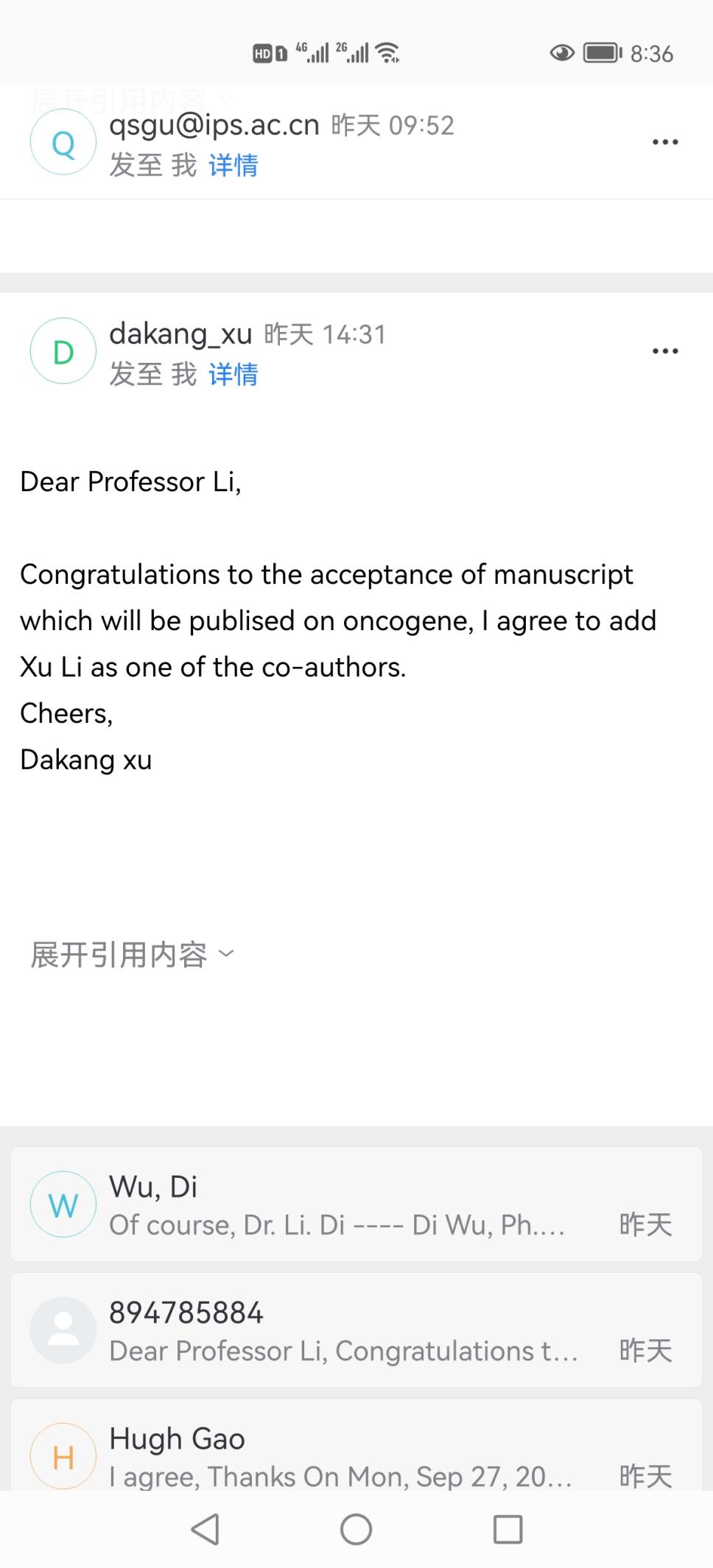

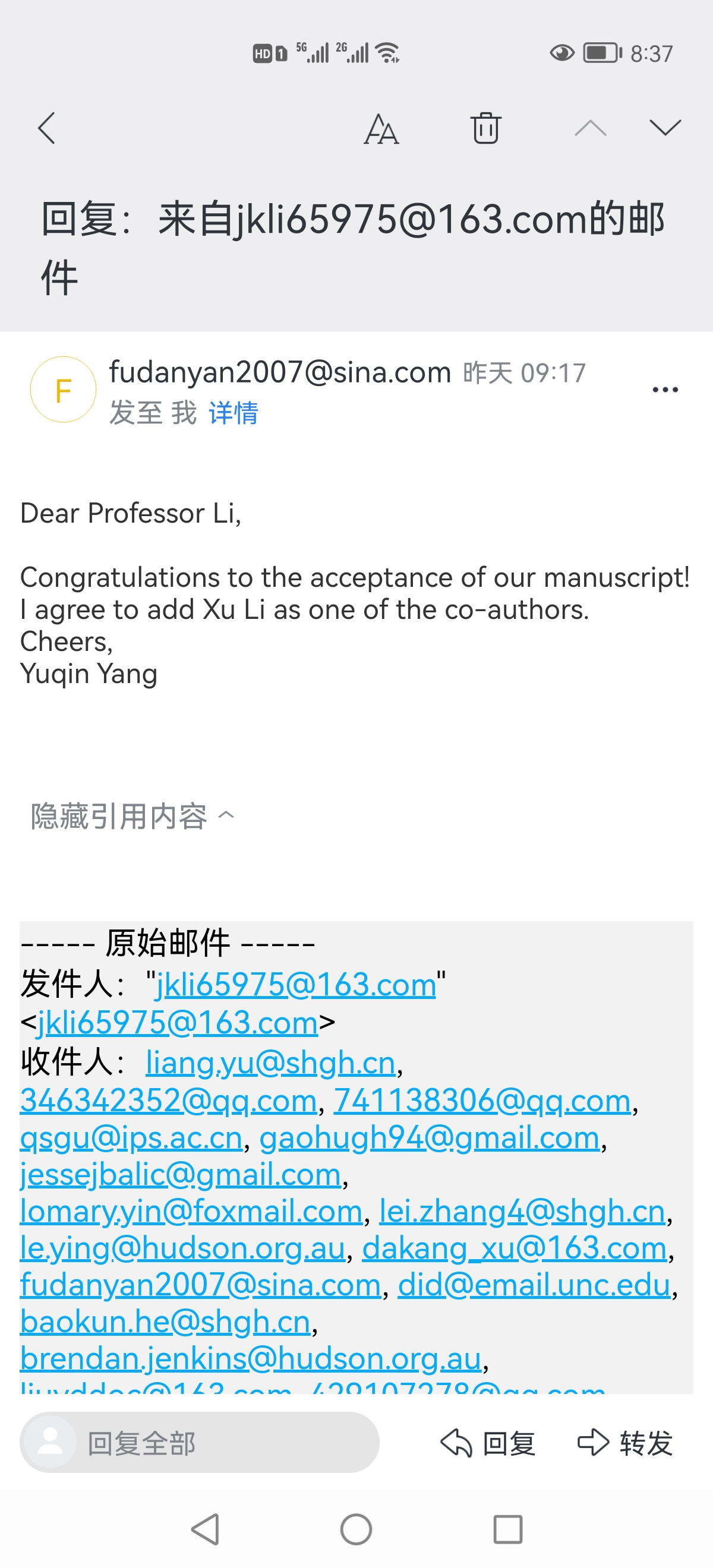

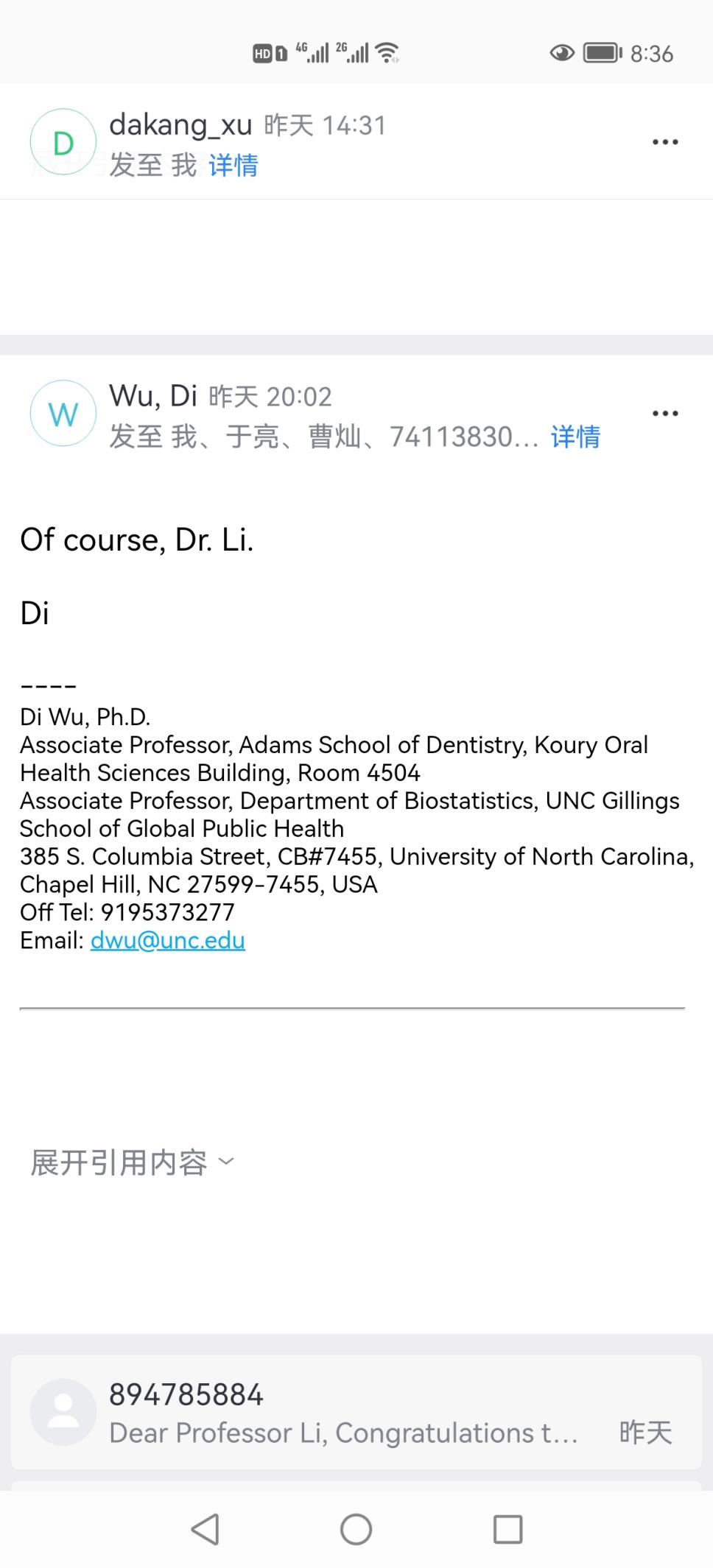

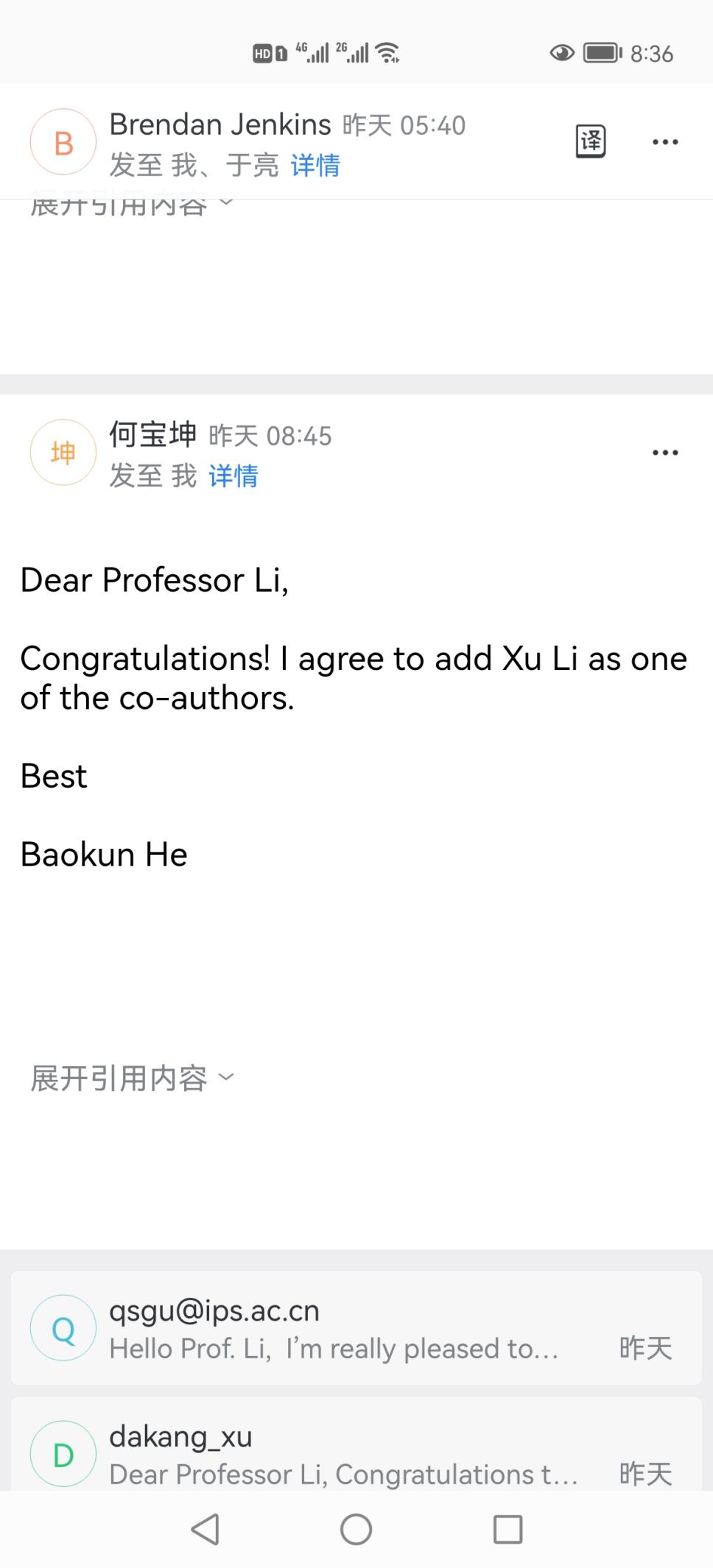

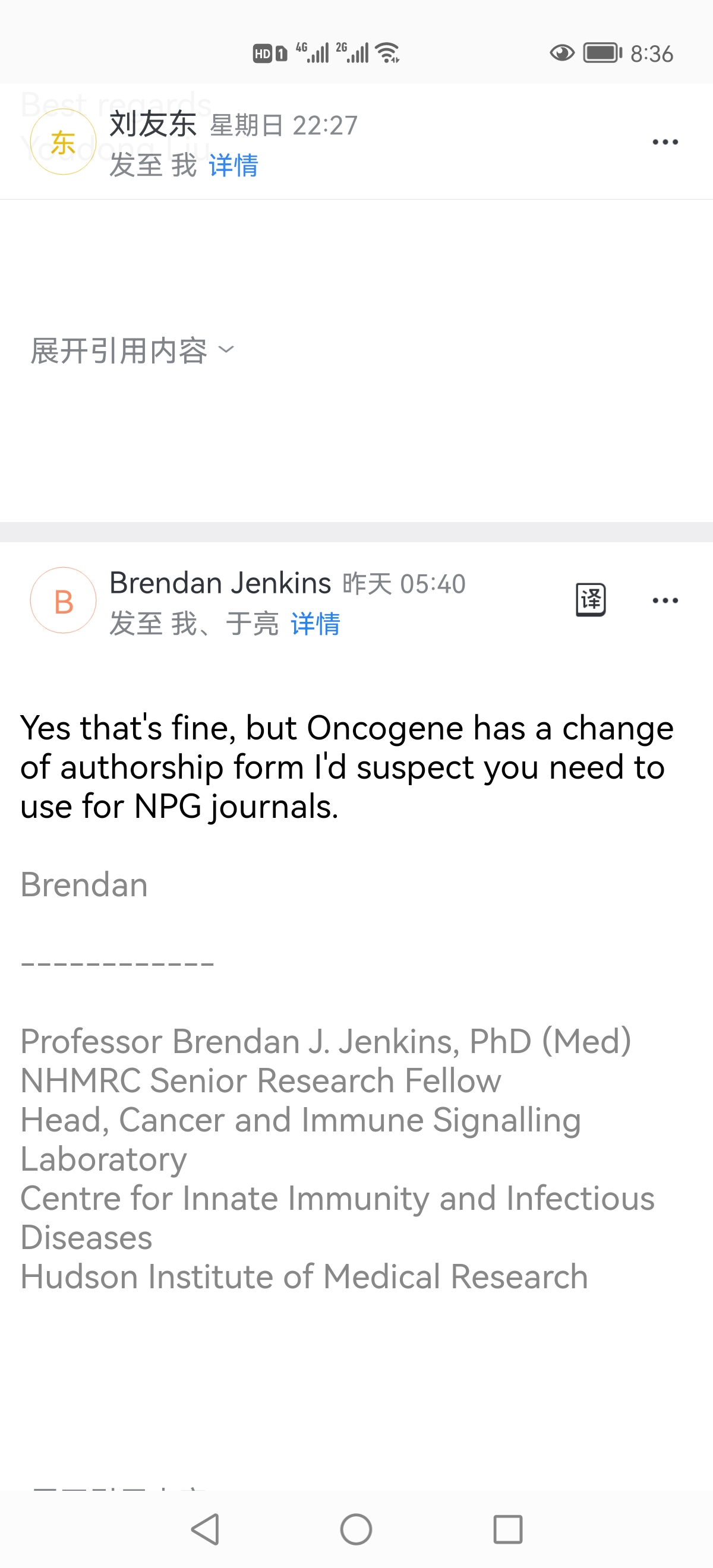

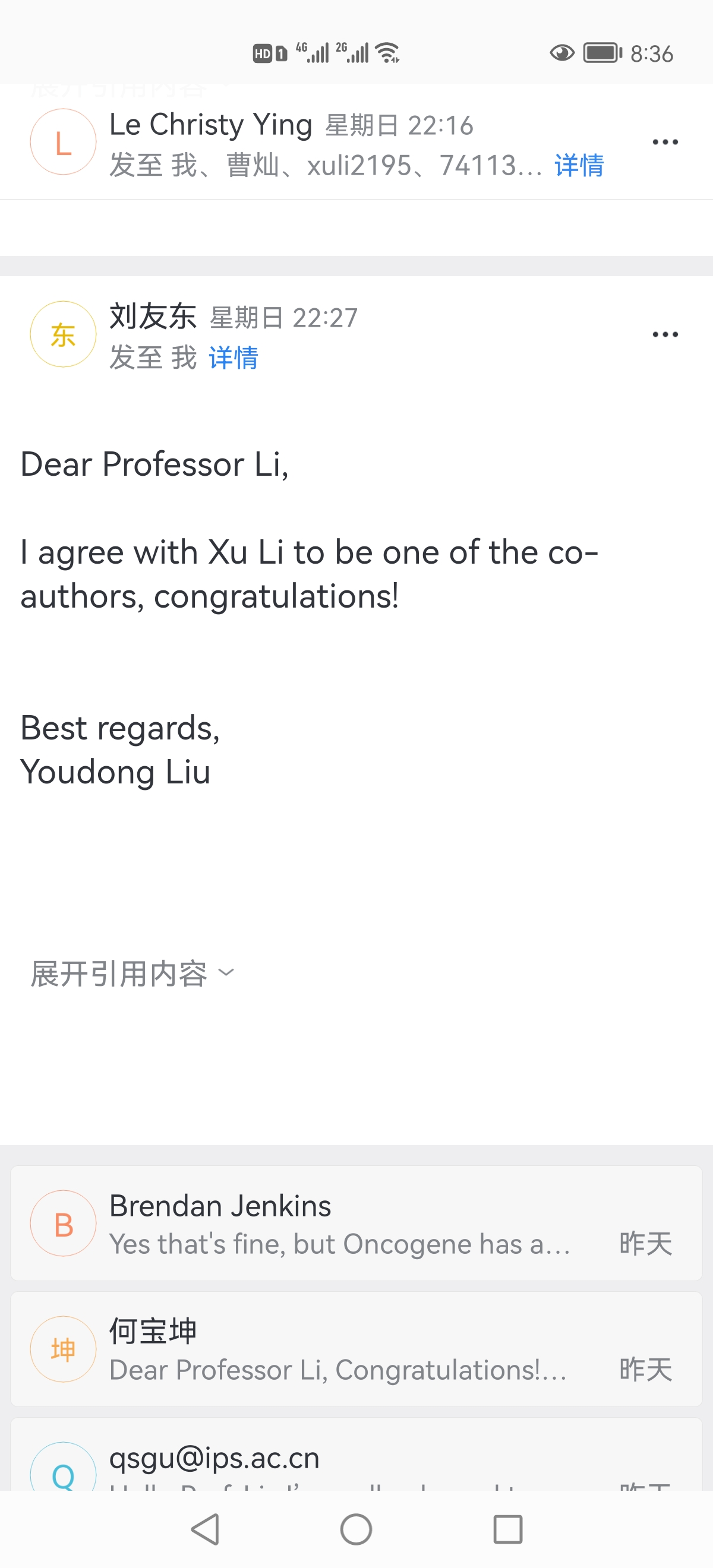

Supplement: Supplementary file 3 — Email communication [file 41388_2021_2067_MOESM3_ESM.docx]
